# Synthesis and molecular structure of novel 2-(alkylthio)-4-chloro-*N*-(4,5-dihydro-5-oxo-1*H*-1,2,4-triazol-3-yl)-5-methylbenzenesulfonamides with potential anticancer activity

**DOI:** 10.1007/s00706-012-0849-7

**Published:** 2012-09-25

**Authors:** Jarosław Sławiński, Beata Żołnowska, Czesława Orlewska, Jarosław Chojnacki

**Affiliations:** 1 Department of Organic Chemistry, Medical University of Gdańsk, Gen. J. Hallera Str. 107, 80416 Gdańsk, Poland; 2 Department of Chemistry, Gdańsk University of Technology, Narutowicza 11/12, 80233 Gdańsk, Poland

**Keywords:** Sulfonamide, Anticancer, Isocyanate, Cyclization, Triazolone

## Abstract

**Abstract:**

A series of novel 4-chloro-*N*-(4,5-dihydro-5-oxo-1-R^2^-1*H*-1,2,4-triazol-3-yl)-5-methyl-2-(R^1^-methylthio)benzenesulfonamide derivatives have been synthesized as potential anticancer agents. The in vitro antitumor activity of some compounds was evaluated in the US National Cancer Institute (NCI) against the NCI-60 cell line panel. The most prominent compound showed remarkable activity against 13 human tumor cell lines representing lung, colon, CNS, melanoma, ovarian, renal, prostate, and breast at low micromolar GI_50_ level in the range of 1.9–3.0 μM.

**Graphical Abstract:**

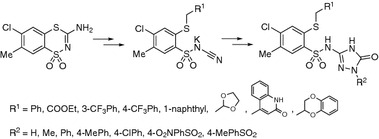

**Electronic supplementary material:**

The online version of this article (doi:10.1007/s00706-012-0849-7) contains supplementary material, which is available to authorized users.

## Introduction

The aryl- and heteroarylsulfonamides are widely described compounds revealing a broad spectrum of applications in biological and pharmacological areas [1]. For many years, 2-mercaptobenzenesulfonamide derivatives (MBSAs) have been of interest because of the various biological properties including antitumor [2–10], antimicrobial [11, 12], and antiviral activities [13, 14], and inhibition of carbonic anhydrase [15–17].

**Fig. 1:**
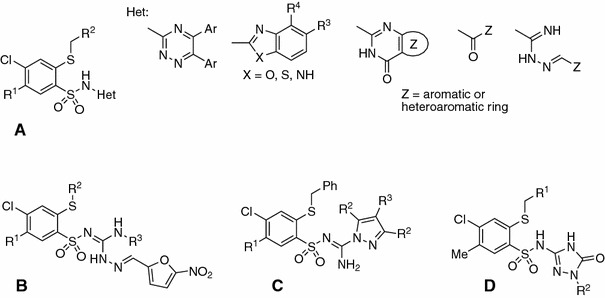
General structures of 2-mercaptobenzenesulfonamides **A**, **B**, **C**, and **D**

It has been known that aryl/heteroarylsulfonamides may act as antitumor agents through a variety of mechanisms such as cell cycle perturbation in the G1 phase, disruption of microtubules, angiogenesis inhibition, and functional suppression of the transcriptional activator NF-Y. The most prominent mechanism was the inhibition of carbonic anhydrase isozymes [18–22]. Recently, a host of structurally novel arylsulfonamide derivatives have been reported to show substantial anticancer activities in vitro and/or in vivo [23–26]. We have reported the synthesis and anticancer activity of 2-mercaptobenzenesulfonamides and subsequently extended our study to analogues with various heterocyclic ring systems attached to the benzenesulfonamide scaffold [4–6, 8, 10, 15] (Fig. 1 structure **A** [4–6, 8, 15], **B** [8], **C** [10]).

In this article we investigated new sulfonamide derivatives containing a triazolone ring in their structure. Triazolones are described in the literature as biologically active compounds, including anti-inflammatories [27], Nk_i_ antagonists [28], inhibitors of tumor necrosis factor-α-converting enzyme (TACE) [29], checkpoint kinase-1 inhibitor [30], anti-tumor agents [31–34], and molecular chaperone Hsp90 inhibitor, which is currently in clinical trials for a number of human cancers [35]. Taking into account the interesting properties of triazolones, we have synthesized novel compounds of general structure **D** (Fig. 1).

## Results and discussion

### Chemistry

**Scheme 1:**
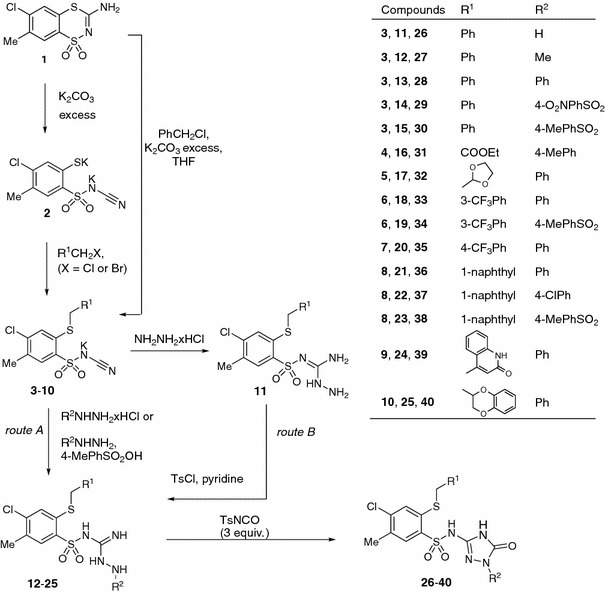

The main goal of this study was to synthesize and investigate the anticancer activity of the new 2-(alkylthio)benzenesulfonamides containing diverse substituted 1,2,4-triazol-5-one moieties. Thus, we propose a synthetic route leading to the target 2-(alkylthio)-4-chloro-*N*-(4,5-dihydro-5-oxo-1-R^2^-1*H*-1,2,4-triazol-3-yl)-5-methylbenzenesulfonamides as shown in Scheme 1.

Starting 3-aminobenzodithiazine **1** could be readily converted to the corresponding dipotassium **2** and potassium salts **3** and **4**, according to the reported procedure for preparation of *N*-(phenylsulfonyl)cyanamide potassium salts [36]. Novel potassium salts **5**–**10** were prepared by the reaction of **2** with the corresponding halomethyl electrophiles such as aryl/cycloalkyl/methyl chlorides in methanol or ethanol. Subsequent reaction of salts **3**–**10** with either hydrazine monohydrochloride, methylhydrazine, *p*-toluenesulfonyl hydrazide, or various 4-substituted phenylhydrazine hydrochlorides led to the formation of the desired 3-(R^2^-amino)-1-[4-chloro-5-methyl-2-(R^1^-methylthio)phenylsulfonyl]guanidine derivatives **11**–**25** as depicted in Scheme 1. It is pertinent to know, however, that aminoguanidine **15** was chosen for the synthesis in two different ways (route A and B in Scheme 1). This was supposed to explain some arising synthetic aspects: whether the usefulness of the potassium salt, i.e., **3** with tosyl hydrazide (route A), is higher than the reaction of aminoguanidine **11** with tosyl chloride (TsCl, route B), and whether the reaction proceeds on the N-terminal nitrogen atom of the sulfonylhydrazide moiety or on the second nitrogen atom neighboring the sulfonyl group. As it turned out, both methods products **15** were identical, with structures (IR, NMR) having a *N’*-substituted sulfonylhydrazide fragment and obtained in almost equal yields.

Many methods are known for the synthesis of 1,2,4-triazol-5-ones. Triazol-5-ones can be prepared for instance by the reaction of the corresponding nitriles via imidates with semicarbazide [37], from 4-substituted semicarbazides under alkaline conditions [32], by heating of *N* ^*1*^,*N* ^*4*^-substituted hydrazinecarboxamides in alkaline media [38], by cyclization of semicarbazide with an excess of phosgene [39], from the reaction of *N*-acylureas with arylhydrazines [40], *N*-acylurethanes with phenylhydrazines as an Einhorn–Brunner reaction extension, as well as from *C*-halobenzylidenephenylhydrazones via nitrilimines with phenyl isocyanates [41, 42].

In the present study we utilized a new method for the synthesis of 1,2,4-triazol-5-ones in the reaction of the corresponding aminoguanudines **11**–**25** with an excess of *p*-toluenesulfonyl isocyanate (TsNCO, Scheme 1). The isocyanates are well known as carbonyl precursors [43] and electrophilic agents whose reactions with hydrazines lead to intramolecular cyclization to five-membered heterocyclic rings [44] or reagents in cycloaddition reactions with various compounds having C=N bonds [45].

Our experiments demonstrated that the proposed synthetic route was an efficient way to prepare the desired *N*-(4,5-dihydro-5-oxo-1*H*-1,2,4-triazol-3-yl)benzenesulfonamides **26**–**40** when an excess of three molar equivalents of tosyl isocyanate was applied in the reaction with the corresponding aminoguanidines **11**–**25** in anhydrous tetrahydrofuran (THF) for at least 9 h at reflux. It is noteworthy, however, when 2 equivalents of tosyl isocyanate were used, no cyclization product was observed and a complex mixture of products was formed, even after considerable extending of the reaction time.

**Fig. 2:**
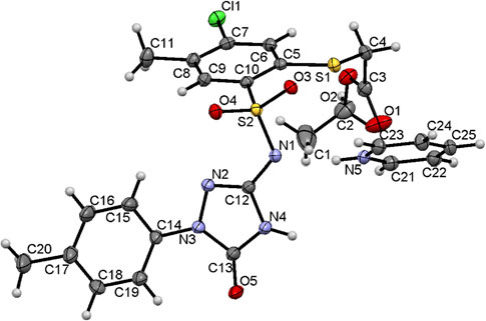
Molecular structure of **31Pyr** showing the atom-labeling scheme. Displacement ellipsoids drawn at the 50 % probability level, with the solvating water molecule omitted

**Fig. 3:**
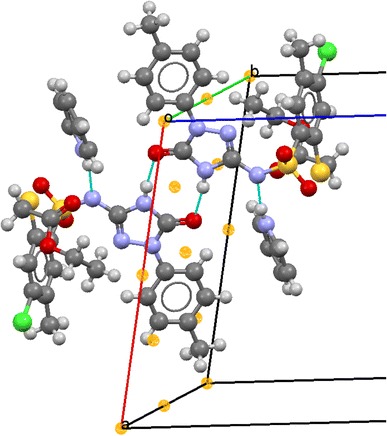
Hydrogen bonds in structure of **31Pyr**. *Blue lines* represent hydrogen bonds; transparent *yellow* balls denote inversion centers in the crystal (generated using Mercury CSD 2.4 [46])

The structure of the new compounds was confirmed by elemental analyses (C, H, N) and spectral (NMR, IR, MS) data presented in the experimental section. Moreover, X-ray analysis was undertaken to confirm proposed structures on the representative compound **31**, which crystallized as pyridinium salt (further specified as **31Pyr**, Figs. 2 and 3).

### Molecular structure

Details on data collection, structure solution, and refinement are given in Table 1. Compound **31Pyr** crystallizes in the monoclinic space group *C*2/*c* with (typical for this symmetry) eight molecules in the unit cell. The molecule, being a secondary benzenesulfonamide, is deprotonated at the N1 atom and in the crystal structure is present in the anionic form (Fig. 2). The proton is accepted by pyridine so a pyridinium ion acts as a counterion. Additionally the solid contains solvating molecules of water that reside on twofold rotation axes, and these positions are not fully occupied by them (s.o.f. = 0.079). Actually, only ca. 1/8 of the H_2_O molecules suffice to fit to the observed electron density in this region.

**Table 1:** Crystal data and structure refinement for compound **31Pyr**

Empirical formula	C_20_H_20_ClN_4_O_5_S_2_·C_5_H_6_N·0.08(O)
Formula weight	577.42
Temperature/K	120(2)
Wavelength/Å	0.71073
Crystal system	Monoclinic
Space group	*C*2/*c*
Unit cell dimensions	
*a*/Å	14.1490(3)
*b*/Å	14.0574(4)
*c*/Å	28.1211(6)
α/°	90
β/°	102.107(2)
γ/°	90
Volume/Å^3^	5,468.8 (2)
*Z*	8
Density (calculated)/mg m^−3^	1.403
Absorption coefficient/mm^−1^	0.338
F(000)	2405
Crystal size/mm^3^	0.20 × 0.13 × 0.10
θ range for data collection/°	2.3–26.0
Index ranges	−17 ≤ h ≤ 17, −12 ≤ k ≤ 17, −25 ≤ l ≤ 34
Reflections collected	12,491
Independent reflections	5,374 [*R*(int) = 0.02]
Completeness to θ = 26.0°	99.6 %
Absorption correction	Numerical
Refinement method	Full-matrix least-squares on *F* ^2^
Data/restraints/parameters	5,374/0/349
Goodness-of-fit on *F* ^2^	1.04
Final *R* indices [*I* > 2σ(*I*)]	*R*_1_ = 0.0405, w*R* ^2^ = 0.1015
*R* indices (all data)	*R*_1_ = 0.0469, w*R* ^2^ = 0.1074
Largest diff. peak and hole/e·Å^−3^	0.43 and −0.31

The two ions are linked by a charge-assisted hydrogen bond of the (+)NH···N(−) type; pyridinium N(5) is a donor, and sulfonamide N(1) is an acceptor. Bonds N(4)–H(4) interact with carbonyl oxygen atoms O5 from the triazolone moiety of the neighboring molecules forming intermolecular hydrogen bonds NH···O. These interactions arranged in pairs can be described by the R_2_^2^(8) motifs situated about local inversion centers (see Fig. 3). Detailed information on hydrogen bonds is given in Table 2. Packing of molecules in the solid state is reinforced also by π–π stacking interactions between adjacent aromatic rings C5–C10 whose centers of gravity (Cg or centroids) are distant at 3.8513(10) Å. The geometry of the interaction is more precisely characterized in Table 3.

**Table 2:** Hydrogen bond geometry in crystal structure of **31Pyr**

D–H···A	D–H/Å	H···A/Å	D···A/Å	D–H···A/°
N4–H4···O5^i^	0.88	1.89	2.765 (2)	171
N5–H5···N1	0.88	1.90	2.767 (2)	169

Symmetry code: (i) −*x* + 1/2, −*y* + 1/2, −*z*

**Table 3:** Main π–π stacking interaction geometry in crystal structure of **31Pyr**

Cg(1)···Cg(1^ii^)^a^/Å	*α* ^b^/°	*β* ^c^/°	Perp.^d^
3.8513(10)	17	15.5	3.7104(7)

Ring (1) is composed from C5–C10 carbon atoms

Symmetry code: (ii) −*x*,* y*, 1/2−*z*

^a^Distance between centroids

^b^Dihedral angle between the rings

^c^Angle between the vector span on the centroids and normal to ring(1)

^d^Perpendicular distance of Cg(I) on the other ring

### Biological assay

Compounds **27**, **28**, and **30**–**39** were initially tested at a single dose (10^−5^ M) in the full NCI-60 cell panel, and the results are shown in Table 4. The methodology of the in vitro cancer screen is described at the website http://www.dtp.nci.nih.gov/branches/btb/ivclsp.html.

**Table 4:** Inhibition growth percent (IGP [%]). One-dose screening data of in vitro tumor growth inhibition for compounds **27**, **28**, **30**–**39** at a dose of 10 μM

Panel	Cell line	Compound											
		**27**	**28**	**30**	**31**	**32**	**33**	**34**	**35**	**36**	**37**	**38**	**39**
Non-small cell	A549/ATCC	26	62	*	*	3	*	2	3	58	*	5	*
Lung cancer	NCI-H522	46	69	71	84	NT	5	5	*	NT	*	83	*
	HOP-92	21	6	29	18	NT	NT	20	NT	NT	5	19	NT
Leukemia	RPMI-8226	36	45	45	39	NT	7	13	11	NT	7	*	*
	SR	7	28	57	31	NT	NT	19	NT	NT	NT	78	NT
	HL-60(TB)	19	13	27	26	*	15	*	NT	91	*	*	22
	MOLT-4	2	10	22	16	*	7	7	22	52	*	21	17
	K-562	14	17	49	37	NT	NT	*	NT	NT	9	66	NT
Renal cancer	RXF 393	2	46	*	1	*	*	*	*	73	2	16	*
	UO-31	15	18	14	26	3	3	11	10	36	24	23	11
CNS cancer	SNB-75	23	*	15	18	7	2	12	10	61	4	19	*
	SF-295	*	*	*	*	*	4	1	2	77	8	3	*
	SF-539	*	*	*	*	*	*	*	*	49	*	*	*
Colon cancer	HCC-2998	1	*	*	*	*	*	*	*	41	*	66	4
	HCT-116	3	6	6	*	*	1	*	4	75	*	62	*
	HCT-15	4	*	7	*	*	5	*	4	65	1	38	2
	HT29	*	*	4	*	*	*	1	*	88	*	53	*
	SW-620	1	*	9	5	*	6	4	*	68	2	55	*
	COLO 205	*	*	*	*	*	NT	*	NT	71	*	NT	NT
	KM12	*	*	5	*	*	*	*	*	79	*	1	*
Breast cancer	MCF7	*	*	38	*	2	*	2	*	83	3	51	*
	T-47D	8	3	20	9	*	*	*	1	31	6	41	1
	MDA-MB-468	NT	NT	NT	NT	*	NT	3	NT	3^a^	NT	61	*
	HS 578T	11	*	24	5	*	NT	2	NT	46	12	*	NT
Ovarian cancer	OVCAR-3	*	1	8	*	*	*	1	*	5^a^	*	65	
	NCI/ADR-RES	NT	NT	NT	NT	*	2	5	9	64	*	32	
Melanoma	UACC-257	*	33	*	*	*	*	4	*	21	*	21	*
	UACC-62	3	7	15	7	*	10	*	7	55	4	*	9
	MALME-3M	5	NT	5	*	*	12	13	4	43	16	25	2
	SK-MEL-2	*	*	*	9	*	*	19	*	26	*	NT	*
	MDA-MB-435	NT	NT	NT	NT	1	9	*	1	29^a^	*	69	*
Prostate cancer	PC-3	9	6	16	8	5	8	5	12	59	*	7	6

Data obtained from NCI-60 DTP human tumor cell line screening

*NT* not tested

* Not active

^a^Cytotoxic effect (lethality)

The relatively highest sensitivity to the compounds described here was found for the cell lines of non-small cell lung cancer NCI-H522 cell line to compounds **27**, **28**, **31**, and **38** (46 % < IGP < 84 %), leukemia RPMI-8226 to compounds **27**, **28**, **30**, and **31** (36 % < IGP < 45 %), HL-60(TB) to **30**, **31**, **36**, and **39** (22 % < IGP < 91 %), and K-562 to compounds **30**, **31**, and **38** (37 % < IGP < 66 %) as well as breast MCF7 to **30**, **36**, and **38** (38 % < IGP < 83 %) (Table 4).

The following conclusions can be drawn from the structure–activity relationship study (Table 4):

1. The susceptibility of the non-small cell lung NCI-H522 cell line against 2-(benzylthio)-*N*-(2,5-dihydro-5-oxo-1-R^2^-1*H*-1,2,4-triazol-3-yl)benzenesulfonamide derivatives (**27**, **28**, **30**) was remarkable and increased when the methyl group (R^2^ = Me, **27**, IGP = 46 %) was replaced by aromatic moieties such as phenyl (R^2^ = Ph, **28**, IGP = 69 %) or tosyl (R^2^ = 4-MePhSO_2_, **30**, IGP = 71 %). The compounds mentioned above showed similar potency for RPMI-8226 (**27**, IGP = 36 %; **28**, IGP = 45 %; **30**, IGP = 45 %) and SR (**27**, IGP = 7 %; **28**, IGP = 28 %; **30**, IGP = 57 %) lines of leukemia. It should be noted, moreover, that replacement of R^2^ = Ph (**28**) for R^2^ = 4-MePhSO_2_ (**30**) caused loss of activity against non-small cell lung cancer (A549/ATCC) and renal (RXF 393) cell lines.
2. For the series of *N*-(4,5-dihydro-5-oxo-1-phenyl-1*H*-1,2,4-triazol-3-yl)-2-(R^1^-methylthio)benzenesulfonamides the substituent at the sulfur atom S-2 at the 2-position of benzenesulfonamide has an impact on the antiproliferative activity against some cancer cell lines: exchange for instance of R^1^ = 1-naphthyl in **36** into R^1^ = Ph (**28**), 1,3-dioxolan-1-yl (**32**), 3-CF_3_Ph (**34**), 4-CF_3_Ph (**35**), and 1,2-dihydro-2-oxoquinolin-4-yl (**39**) decreased activity against the leukemia HL-60(TB) cell line, as well as the leukemia MOLT-4 cell line; replacing R^1^ = 1-naphthyl or Ph for R^1^ = 3-CF_3_Ph, 4-CF_3_Ph or 1,2-dihydro-2-oxoquinolin-4-yl resulted in loss of activity against cell lines non-small cell lung A549/ATCC and renal RXF 393.
3. The significant susceptibility of almost the entire colon cancer subpanel against *N*-(4,5-dihydro-5-oxo-1-R^2^-1*H*-1,2,4-triazol-3-yl)-2-(naphthalen-1-ylmethylthio)benzenesulfonamides **36** and **38** should be pointed out. Moreover, the exchange of R^2^ = Ph (**36**) or 4-MePhSO_2_ (**38**) for 4-ClPh (**37**) led to a lack of susceptibility of HCC-2998, HTC-116, HTC-15, HT29, and SW-620.

Further anticancer evaluation was performed at five-dose assay on the distinctive compound **36**. The anticancer activity of the tested compound was reported for each cell line by the parameters GI_50_ (molar concentration of the compounds that inhibit 50 % net cell growth), TGI (molar concentration of the compounds leading to total inhibition), and LC_50_ (molar concentration of the compounds causing 50 % net cell death). The susceptibility of individual subpanels indicates the following order: prostate, colon, CNS, leukemia, ovarian, non-small cell lung, melanoma, renal, and breast cancer (Table 5). As shown in Table 5, compound **36** exhibited remarkable activity at low GI_50_ level <11.2 μM (MID GI_50_ = 4.2 μM) over a number of cancer cell lines, acting effectively against 13 human tumor cell lines with GI_50_ values in the low micromolar range of 1.9–3.0 μM with selectivity toward melanoma MDA-MB-435 (GI_50_ = 1.9 μM, TGI = 5.5) and renal A498 (GI_50_ = 1.9 μM, TGI = 10.5) cell lines. It is worth mentioning that lines HL-60(TB), NCI-H522, COLO 205, SF-539, MDA-MB-435, OVCAR-3, A498, RXF 393, DU-145, and MDA-MB-468 were characterized by the relatively low parameters GI_50_ (1.9–3.2 μM), TGI (4.9–12.3 μM), and LC_50_ below 58.7 μM.

**Table 5:** The in vitro tumor growth inhibition data for compound **36**

Panel	Cell line	GI_50_^a^/μM	TGI^b^/μM	LC_50_^c^/μM
Leukemia	CCRF-CEM	3.2	13.2	>100
	HL-60(TB)	3.1	9.2	58.7
	K-562	3.6	13.9	>100
	MOLT-4	5.7	37.2	>100
	RPMI-8226	3.6	28.1	>100
Non-small cell lung cancer	A549/ATCC	4.4	27.8	>100
	EKVX	4.6	23.3	>100
	HOP-62	7.1	20.8	49.3
	HOP-92	7.5	23.0	57.0
	NCI-H226	3.6	16.1	53.0
	NCI-H23	3.1	11.7	42.8
	NCI-H322M	8.3	32.9	>100
	NCI-H460	2.8	11.7	>100
	NCI-H522	2.5	8.3	44.1
Colon cancer	COLO 205	2.3	4.9	11.1
	HCC-2998	3.5	12.6	40.0
	HCT-116	3.4	12.6	43.0
	HCT-15	4.0	15.3	47.8
	HT29	3.5	11.7	41.9
	KM12	3.2	12.5	46.1
	SW-620	4.6	18.6	48.9
CNS cancer	SF-268	3.7	15.5	48.2
	SF-295	3.0	13.9	>100
	SF-539	3.2	9.4	44.1
	SNB-19	4.8	23.5	95.3
	SNB-75	2.9	15.5	85.1
	U251	5.3	19.9	54.9
Melanoma	LOX IMVI	4.8	18.3	48.8
	MALME-3 M	8.9	29.4	92.2
	M14	3.2	11.5	64.9
	MDA-MB-435	1.9	5.5	32.0
	SK-MEL-2	5.8	20.7	60.2
	SK-MEL-28	6.0	20.1	54.2
	SK-MEL-5	3.6	13.1	36.6
	UACC-257	7.9	33.6	>100
	UACC-62	4.0	16.1	48.5
Ovarian cancer	IGROV1	4.9	21.9	97.0
	OVCAR-3	2.4	6.2	23.6
	OVCAR-4	4.2	16.5	55.1
	OVCAR-5	6.3	19.4	46.8
	OVCAR-8	5.0	33.0	>100
	NCI/ADR-RES	2.7	8.6	>100
	SK-OV-3	3.9	18.1	>100
Renal cancer	786-0	7.9	22.0	54.3
	A498	1.9	10.5	38.0
	ACHN	5.8	21.9	68.7
	CAKI-1	4.2	25.9	>100
	RXF 393	2.5	7.6	32.2
	SN12C	4.6	18.2	52.7
	TK-10	10.3	24.2	56.8
	UO-31	5.5	18.7	45.9
Prostate cancer	PC-3	3.6	17.1	73.7
	DU-145	2.9	8.6	32.6
Breast cancer	MCF7	3.8	15.3	>100
	MDA-MB-231/ATCC	5.6	20.5	60.4
	HS 578T	2.9	19.6	>100
	BT-549	11.2	26.0	60.4
	T-47D	6.1	35.9	>100
	MDA-MB-468	3.0	12.3	49.9

Data obtained from NCI-60 DTP human tumor cell line screening

^a^GI_50_: molar concentration that inhibits 50 % net cell growth

^b^TGI: molar concentration giving total growth inhibition

^c^LC_50_: molar concentration causing 50 % net cell death

A COMPARE [47] analysis at the NCI of compound **36** showed a moderate Pearson’s correlation coefficient (PCC = 0.473–0.425) with agents disrupting microtubule formation such as maytansine and rhizoxin [48].

## Conclusion

We have developed a new method for the synthesis of a series of 2-(alkylthio)-4-chloro-*N*-(4,5-dihydro-5-oxo-1-R^2^-1*H*-1,2,4-triazol-3-yl)-5-methylbenzenesulfonamides **26**–**40**. The prominent compound **36** showed high (GI_50_ = 1.9–3.0 μM) activity against 13 of the tumor cell lines and reasonable activity at level GI_50_ <11.2 μM (MID GI_50_ = 4.2 μM) over a number cell lines, suggesting that **36** may be a useful lead compound for the search for more powerful anticancer agents with low toxicity against normal cells.

## Experimental

The following instruments and parameters were used: melting points: Boetius apparatus; IR spectra: KBr pellets, 400–4,000 cm^−1^, Thermo Mattson Satellite FTIR spectrometer; ^1^H NMR and ^13^C NMR: Varian Gemini 200 apparatus or Varian Unity Plus 500 MHz, chemical shifts are expressed as δ values relative to Me_4_Si as standard; LC–MS analyses: Shimadzu LCMS-IT-TOF LC-20A mass spectrometer with an electrospray ionization, capillary voltage in positive ion mode +4.5 kV, column: Jupiter 4 u Proteo 90 Å, 4.0 × 150 mm, 4 μm, mobile phase: A—water with 0.1 % formic acid, B—0.1 % formic acid in acetonitrile, linear gradient 50–100 % B in 45 min, flow rate: 0.2 cm^3^ min^−1^. The results of elemental analyses for C, H, and N were in agreement with the calculated values within ±0.3 % range. Thin-layer chromatography (TLC) was performed on Merck Kieselgel 60F254 plates and visualized with UV. *N*-(5-Methylphenylsulfonyl)cyanamide potassium salts **3**, **4** and aminoguanidines **11**–**14** and **16** were obtained in accordance with the previously described procedures [2, 36].

### *N*-*[4*-*Chloro*-*2*-*(1,3*-*dioxolan*-*2*-*ylmethylthio)*-*5*-*methylphenylsulfonyl]cyanamide potassium salt* (**5**, C_12_H_12_ClKN_2_O_4_S_2_)

To a suspension of 3.05 g 5-chloro-2-(cyanoaminosulfonyl)-4-methylthiophenolate dipotassium salt (**2**, 9 mmol) in 9 cm^3^ methanol 2.4 cm^3^ 2-(bromomethyl)-1,3-dioxolane (23 mmol) was added dropwise for 5 min. The reaction mixture was stirred at 65 °C for 6.5 h, then 12 h at room temperature. The precipitate was collected by filtration. The filtrate was evaporated to dryness, and the residue was triturated with 90 cm^3^ diethyl ether to give a second fraction of precipitate. The product was extracted from the combined fractions of solid with hot ethanol to give 2.99 g (86 %) **5**. M.p.: 224–225 °C; TLC: *R*_f_ = 0.74 (CHCl_3_:MeOH = 3:1); IR (KBr): $\bar{v}$ = 2,924 (CH_3_, CH_2_), 2,854 (CH_3_, CH_2_), 2,179 (C≡N), 1,339, 1,145 (SO_2_) cm^−1^; ^1^H NMR (200 MHz, DMSO-*d*_*6*_): δ = 2.31 (s, 3H, CH_3_), 3.21 (d, 2H, S–CH_2_), 3.79–4.00 (m, 4H, CH_2_–O), 5.11 (t, 1H, CH–O), 7.46 (s, 1H, H-3), 7.77 (s, 1H, H-6) ppm; ^13^C NMR (50 MHz, DMSO-*d*_*6*_): δ = 19.22, 36.04, 64.96, 102.34, 117.50, 127.42, 130.74, 131.36, 135.75, 135.97, 140.86 ppm.

### General procedure for the preparation of N-[4-chloro-5-methyl-2-(R^1^-methylthio)phenylsulfonyl]cyanamide potassium salts** 6**–**10**

To a suspension of 3.05 g 5-chloro-2-(cyanoaminosulfonyl)-4-methylthiophenolate dipotassium salt (**2**, 9 mmol) in methanol or ethanol the appropriate halomethyl electrophile was added. The reaction mixture was stirred at room temperature or at 65 °C. The precipitate was collected by filtration. The product was separated from inorganic salts by extraction with 200 cm^3^ hot ethanol.

#### *N*-*[4*-*Chloro*-*5*-*methyl*-*2*-*[3*-*(trifluoromethyl)benzylthio]phenylsulfonyl]cyanamide potassium salt* (**6**, C_16_H_11_ClF_3_KN_2_O_2_S_2_)

Starting from **2** in 45 cm^3^ ethanol and 1.3 cm^3^ 3-(trifluoromethyl)benzyl chloride (9 mmol) for 2 h at room temperature, compound **6** was obtained. Yield: 3.46 g (84 %); m.p.: 158–160 °C; TLC: *R*_f_ = 0.87 (CHCl_3_:MeOH = 2:1); IR (KBr): $\bar{v}$ = 2,924 (CH_3_, CH_2_), 2,174 (C≡N), 1,332, 1,132 (SO_2_) cm^−1^; ^1^H NMR (200 MHz, DMSO-*d*_*6*_): δ = 2.31 (s, 3H, CH_3_), 4.41 (s, 2H, S–CH_2_), 7.41 (s, 1H, H-3), 7.58-7.62 (m, 2H, Ar), 7.76–7.81 (m, 3H, H-6, Ar) ppm; ^13^C NMR (50 MHz, DMSO-*d*_*6*_): δ = 19.23, 35.73, 117.46, 124.12, 124.20, 125.95, 126.03, 127.78, 129.77, 130.85, 131.89, 133.49, 134.82, 135.94, 138.57, 141.12 ppm.

#### *N*-*[4*-*Chloro*-*5*-*methyl*-*2*-*[4*-*(trifluoromethyl)benzylthio]phenylsulfonyl]cyanamide potassium salt* (**7**, C_16_H_11_ClF_3_KN_2_O_2_S_2_)

Starting from **2** in 45 cm^3^ ethanol and 1.3 cm^3^ 4-(trifluoromethyl)benzyl chloride (9 mmol) for 4 h at room temperature, compound **7** was obtained. Yield: 3.64 g (88 %); m.p.: 177–178 °C; TLC: *R*_f_ = 0.69 (ethyl acetate:isopropanol = 2:1); IR (KBr): $\bar{v}$ = 2,921 (CH_3_, CH_2_), 2,176 (C≡N), 1,327, 1,137 (SO_2_) cm^−1^; ^1^H NMR (500 MHz, DMSO-*d*_*6*_): δ = 2.29 (s, 3H, CH_3_), 4.38 (s, 2H, S–CH_2_), 7.38 (s, 1H, H-3), 7.66 (d, 2H, Ar), 7.68 (d, 2H, Ar), 7.73 (s, 1H, H-6) ppm; ^13^C NMR (50 MHz, DMSO-*d*_*6*_): δ = 19.23, 35.78, 117.50, 125.41, 125.49, 125.56, 127.57, 130.13, 130.87, 131.84, 134.93, 135.96, 141.06, 142.02 ppm.

#### *N*-*[4*-*Chloro*-*5*-*methyl*-*2*-*(naphthalen*-*1*-*ylmethylthio)phenylsulfonyl]cyanamide potassium salt* (**8**, C_19_H_14_ClKN_2_O_2_S_2_)

Starting from **2** in 10 cm^3^ ethanol and 1.3 cm^3^ 1-(chloromethyl)naphthalene (9 mmol) for 1 h at room temperature, compound **8** was obtained. Yield: 3.09 g (78 %); m.p.: 223–225 °C; TLC: *R*_f_ = 0.63 (ethyl acetate:isopropanol = 2:1); IR (KBr): $\bar{v}$ = 2,922 (CH_3_, CH_2_), 2,175 (C≡N), 1,341, 1,140 (SO_2_) cm^−1^; ^1^H NMR (500 MHz, DMSO-*d*_*6*_): δ = 2.32 (s, 3H, CH_3_), 4.72 (s, 2H, S–CH_2_), 7.44–7.47 (m, 1H, Ar), 7.51 (s, 1H, H-3), 7.52–7.59 (m, 2H, Ar), 7.61 (d, 1H, Ar), 7.76 (s, 1H, H-6), 7.87 (d, 1H, Ar), 7.95 (d, 1H, Ar), 8.24 (d, 1H, Ar) ppm; ^13^C NMR (50 MHz, DMSO-*d*_*6*_): δ = 19.29, 34.68, 117.49, 124.62, 125.78, 126.20, 126.52, 127.76, 128.20, 128.36, 128.76, 130.80, 131.60, 131.71, 132.20, 133.68, 136.04, 136.15, 140.76 ppm.

#### *N*-*[4*-*Chloro*-*2*-*(1,2*-*dihydro*-*2*-*oxoquinolin*-*4*-*ylmethylthio)*-*5*-*methylphenylsulfonyl]cyanamide potassium salt* (**9**, C_18_H_13_ClKN_3_O_3_S_2_)

Starting from **2** in 42 cm^3^ ethanol and 2.1 g 4-(bromomethyl)quinolin-2(1*H*)-one (9 mmol) for 4 h at room temperature, compound **9** was obtained. Yield: 3.60 g (88 %); m.p.: 199–201 °C; TLC: *R*_f_ = 0.61 (ethyl acetate:isopropanol:acetic acid = 1:1:0.02); IR (KBr): $\bar{v}$ = 2,922 (CH_3_, CH_2_), 2,181 (C≡N), 1,668 (CO), 1,341, 1,142 (SO_2_) m^−1^; ^1^H NMR (500 MHz, DMSO-*d*_*6*_): δ = 2.31 (s, 3H, CH_3_), 4.52 (s, 2H, S-CH_2_), 6.57 (s, 1H, Ar), 7.21 (t, 1H, Ar), 7.32 (d, 1H, Ar), 7.40 (s, 1H, H-3), 7.51 (t, 1H, Ar), 7.77 (s, 1H, H-6), 7.93 (d, 1H, Ar), 11.74 (s, 1H, NH) ppm; ^13^C NMR (50 MHz, DMSO-*d*_*6*_): δ = 19.30, 33.33, 115.88, 117.50, 118.47, 121.99, 122.08, 125.42, 128.14, 130.82, 132.21, 134.60, 136.04, 139.22, 141.24, 146.29, 161.59 ppm.

#### *N*-*[4*-*Chloro*-*2*-*(2,3*-*dihydrobenzo[b][1,4]dioxin*-*2*-*ylmethylthio)*-*5*-*methylphenylsulfonyl]cyanamide potassium salt* (**10**, C_17_H_14_ClKN_2_O_4_S_2_)

Starting from **2** in 23 cm^3^ methanol and 1.7 cm^3^ 2-(bromomethyl)-1,4-benzodioxane (12 mmol) for 6 h at 65 °C, compound **10** was obtained. Yield: 3.2 g (78 %); m.p.: 98–100 °C; TLC: *R*_f_ = 0.86 (CHCl_3_:pentane:acetone = 1:1:0.5); IR (KBr): $\bar{v}$ = 2,923 (CH_3_, CH_2_), 2,176 (C≡N), 1,343, 1,143 (SO_2_) cm^−1^; ^1^H NMR (200 MHz, DMSO-*d*_*6*_): δ = 2.33 (s, 3H, CH_3_), 3.34 (d, 2H, S–CH_2_), 4.04–4.13 (m, 2H, CH_2_–O), 4.29–4.43 (m, 1H, CH–O), 6.80–6.91 (m, 4H, Ar), 7.57 (s, 1H, H-3), 7.78 (s, 1H, H-6) ppm; ^13^C NMR (50 MHz, DMSO-*d*_*6*_): δ = 19.30, 32.96, 66.34, 71.91, 107.38, 117.23, 117.37, 121.61, 121.79, 128.34, 130.85, 132.24, 134.60, 136.20, 141.72, 142.81, 143.13 ppm.

### General procedure for the preparation of 1-[4-chloro-5-methyl-2-(R^1^-methylthio)phenylsulfonyl]-3-(R^2^-amino)guanidines** 15**,** 17**–**25**

To a suspension of the appropriate *N*-(phenylsulfonyl)cyanamide potassium salt (**3**, **5**–**10**, 3.5 mmol) in dry toluene was added the corresponding phenylhydrazine hydrochloride derivative (3.5 mmol) or *p*-toluenesulfonyl hydrazide (3.5 mmol) in the presence of *p*-toluenesulfonic acid monohydrate (PTSA, 3.5 mmol). The reaction mixture was stirred at reflux for 1–8 h, and left overnight at 0 °C. The precipitate was filtered off, and dried, then treated with 20 cm^3^ of water. After vigorously stirring for 30 min the precipitate was collected by filtration, dried, and crystallized from ethanol (**15**, **17**, **19**, **21**-**23**, **25**), ethyl acetate/hexane (**18**), or ethyl acetate (**20**, **24**).

#### *1*-*[2*-*(Benzylthio)*-*4*-*chloro*-*5*-*methylphenylsulfonyl]*-*3*-*(4*-*methylphenylsulfonylamino)guanidine* (**15**, C_22_H_23_ClN_4_O_4_S_3_)

*Method A.* According to the general procedure, starting from 1.37 g **3**, 0.65 g *p*-toluenesulfonyl hydrazide, and 0.66 g PTSA in 40 cm^3^ of dry toluene for 1 h, the title compound **15** was obtained. Yield: 1.62 g (86 %); m.p.: 242–244 °C; TLC: *R*_f_ = 0.38 (CHCl_3_:pentane:acetone = 1:1:0.5); IR (KBr): $\bar{v}$ = 3,469, 3,361 (NH), 2,922, 2,832 (CH_3_, CH_2_), 1,384, 1,340, 1,172, 1,141 (SO_2_) cm^−1^; ^1^H NMR (500 MHz, DMSO-*d*_*6*_): δ = 2.30 (s, 3H, CH_3_), 2.39 (s, 3H, CH_3_), 4.29 (s, 2H, S–CH_2_), 7.18 (brs, 1H, NH=), 7.25–7.28 (m, 1H, Ar), 7.32–7.38 (m, 4H, Ar), 7.42–7.43 (m, 4H, H-3, NH, Ar), 7.66 (d, 2H, Ar), 7.79 (s, 1H, H-6), 9.23 (s, 1H, N–NHSO_2_), 9.89 (s, 1H, SO_2_NH) ppm; ^13^C NMR (50 MHz, DMSO-*d*_*6*_): δ = 19.20, 21.35, 36.60, 127.51, 128.04, 128.15, 128.74, 129.41, 129.89, 130.58, 132.09, 134.71, 135.86, 136.48, 136.74, 139.64, 144.17, 158.47 ppm.

*Method B.* To a cooled mixture of 1.35 g **11** (3.5 mmol) in 5 cm^3^ dry pyridine was added 0.67 g tosyl chloride (3.5 mmol). The ice bath was removed and the mixture was stirred at room temperature for 4 h, then at 60–65 °C for 5 h. After standing overnight, the mixture was added dropwise to 12 cm^3^ slush and vigorously stirred for 2 h. The solid was filtered off, washed with water (5 × 20 cm^3^), 1 % HCl (2 × 20 cm^3^), water (2 × 20 cm^3^) and dried. Purification from MeOH yielded **15** (86 %); m.p. 242–244 °C (dec.); IR and ^1^H NMR spectra were identical with an authentic sample of **15**.

#### *1*-*[4*-*Chloro*-*2*-*(1,3*-*dioxolan*-*2*-*ylmethylthio)*-*5*-*methylphenylsulfonyl]*-*3*-*(phenylamino)guanidine* (**17**, C_18_H_21_ClN_4_O_4_S_2_)

Starting from 1.36 g **5** and 0.51 g phenylhydrazine hydrochloride in 5 cm^3^ dry toluene for 1 h, the title compound **17** was obtained. Yield: 0.80 g (50 %); m.p.: 173–175 °C; TLC: *R*_f_ = 0.59 (CHCl_3_:pentane:acetone = 1:1:0.5); IR (KBr): $\bar{v}$ = 3,447 (NH), 2,923 (CH_3_, CH_2_), 1,393, 1140 (SO_2_) cm^−1^; ^1^H NMR (500 MHz, DMSO-*d*_*6*_): δ = 2.32 (s, 3H, CH_3_), 3.28 (d, 2H, S-CH_2_), 3.79–3.85 (m, 2H, CH_2_–O), 3.92–3.98 (m, 2H, CH_2_–O), 5.14 (t, 1H, CH–O), 6.69 (d, 2H, Ar), 6.79 (t, 1H, Ar), 7.02 (s, 1H, NH=), 7.17 (t, 2H, Ar), 7.38 (s, 1H, NH-Ph), 7.56 (s, 1H, H-3), 7.86 (s, 1H, H-6), 7.88 (s, 1H, N*H*-NH-Ph), 9.07 (s, 1H, NHSO_2_) ppm; ^13^C NMR (50 MHz, DMSO-*d*_*6*_): δ = 19.22, 36.44, 65.06, 102.35, 112.99, 120.01, 128.16, 129.09, 130.56, 132.04, 136.00, 136.64, 140.28, 148.23, 159.25 ppm.

#### *1*-*[4*-*Chloro*-*5*-*methyl*-*2*-*[3*-*(trifluoromethyl)benzylthio]phenylsulfonyl]*-*3*-*(phenylamino)guanidine* (**18**, C_22_H_20_ClF_3_N_4_O_2_S_2_)

Staring from 1.61 g **6** and 0.50 g phenylhydrazine hydrochloride in 11 cm^3^ dry toluene for 1 h, the title compound **18** was obtained. Yield: 1.26 g (68 %); m.p.: 184–185 °C; TLC: *R*_f_ = 0.67 (CHCl_3_:pentane:acetone = 1:1:0.5); IR (KBr): $\bar{v}$ = 3,444 (NH), 2,925 (CH_3_, CH_2_), 1,330, 1,120 (SO_2_) cm^−1^; ^1^H NMR (200 MHz, DMSO-*d*_*6*_): δ = 2.30 (s, 3H, CH_3_), 4.46 (s, 2H, S–CH_2_), 6.66 (d, 2H, Ar), 6.77 (t, 1H, Ar), 7.04 (s, 1H, NH=), 7.13 (t, 2H, Ar), 7.40 (s, 1H, NH-Ph), 7.47 (s, 1H, H-3), 7.55–7.68 (m, 2H, Ar), 7.74 (s, 1H, H-6), 7.97 (s, 1H, N*H*-NH-Ph), 7.88–7.97 (m, 2H, Ar), 9.08 (s, 1H, NHSO_2_) ppm; ^13^C NMR (50 MHz, DMSO-*d*_*6*_): δ = 19.22, 35.90, 112.94, 119.97, 124.20, 124.27, 126.01, 126.08, 128.42, 129.05, 129.89, 130.77, 132.49, 133.44, 134.90, 136.59, 138.39, 140.47, 148.16, 159.19 ppm.

#### *1*-*[4*-*Chloro*-*5*-*methyl*-*2*-*[3*-*(trifluoromethyl)benzylthio]phenylsulfonyl]*-*3*-*(4*-*methylphenylsulfonylamino)guanidine* (**19**, C_23_H_22_ClF_3_N_4_O_4_S_3_)

Starting from 1.61 g **6**, 0.65 g *p*-toluenesulfonyl hydrazide, and 0.66 g PTSA in 40 cm^3^ dry toluene for 1.5 h, the title compound **19** was obtained. Yield: 1.49 g (70 %); m.p.: 190–191 °C; TLC: *R*_f_ = 0.65 (CHCl_3_:MeOH = 16:3); IR (KBr): $\bar{v}$ = 3,459, 3,360, 3,310 (NH), 2,926 (CH_3_, CH_2_), 1,635 (C=N), 1,333, 1,174, 1,126 (SO_2_) cm^−1^; ^1^H NMR (200 MHz, DMSO-*d*_*6*_): δ = 2.29 (s, 3H, CH_3_), 2.38 (s, 3H, CH_3_), 4.40 (s, 2H, S–CH_2_), 7.24 (brs, 1H, NH), 7.36 (d, 2H, Ar tosyl), 7.43 (s, 1H, NH), 7.50–7.77 (m, 7H, Ar, Ar tosyl), 7.78 (s, 1H, H-6), 9.22 (s, 1H, SO_2_NH), 9.90 (s, 1H, SO_2_NH) ppm; ^13^C NMR (50 MHz, DMSO-*d*_*6*_): δ = 19.22, 21.32, 36.01, 124.31, 124.39, 126.00, 126.08, 128.13, 128.89, 129.51, 129.89, 130.59, 132.68, 133.46, 134.69, 134.73, 136.69, 138.41, 140.23, 144.18, 158.46 ppm.

#### *1*-*[4*-*Chloro*-*5*-*methyl*-*2*-*[4*-*(trifluoromethyl)benzylthio]phenylsulfonyl]*-*3*-*(phenylamino)guanidine* (**20**, C_22_H_20_ClF_3_N_4_O_2_S_2_)

Starting from 1.61 g **7** and 0.51 g phenylhydrazine hydrochloride in 13 cm^3^ dry toluene for 2 h, the title compound **20** was obtained. Yield: 1.04 g (56 %); m.p.: 161–164 °C; TLC: *R*_f_ = 0.71 (CHCl_3_:pentane:acetone = 1:1:0.5); IR (KBr): $\bar{v}$ = 3,433 (NH), 2,924 (CH_3_, CH_2_), 1,325, 1,129 (SO_2_) cm^−1^; ^1^H NMR (500 MHz, DMSO-*d*_*6*_): δ = 2.29 (s, 3H, CH_3_), 4.46 (s, 2H, S–CH_2_), 6.65 (d, 2H, Ar), 6.76 (t, 1H, Ar), 7.04 (s, 1H, NH=), 7.11 (t, 2H, Ar), 7.40 (s, 1H, NH-Ph), 7.46 (s, 1H, H-3), 7.62–7.72 (m, 4H, H-6, Ar, N*H*-NH-Ph), 7.89 (d, 2H, Ar), 9.06 (s, 1H, NHSO_2_) ppm; ^13^C NMR (50 MHz, DMSO-*d*_*6*_): δ = 19.21, 35.81, 112.94, 119.96, 125.48, 125.56, 125.63, 128.03, 129.04, 130.12, 130.79, 132.39, 135.04, 136.65, 140.29, 141.74, 148.15, 159.21 ppm.

#### *1*-*[4*-*Chloro*-*5*-*methyl*-*2*-*(naphthalen*-*1*-*ylmethylthio)phenylsulfonyl]*-*3*-*(phenylamino)guanidine* (**21**, C_25_H_23_ClN_4_O_2_S_2_)

Starting from 1.54 g **8** and 0.5 g phenylhydrazine hydrochloride in 10 cm^3^ dry toluene for 1 h, the title compound **21** was obtained. Yield: 0.82 g (40 %); m.p.: 145–150 °C; TLC: *R*_f_ = 0.71 (CHCl_3_:pentane:acetone = 1:1:0.5); IR (KBr): $\bar{v}$ = 3,331 (NH), 2,922 (CH_3_, CH_2_), 1,391, 1,137 (SO_2_) cm^−1^; ^1^H NMR (200 MHz, DMSO-*d*_*6*_): δ = 2.33 (s, 3H, CH_3_), 4.79 (s, 2H, S–CH_2_), 6.62 (d, 2H, Ar), 6.75 (t, 1H, Ar), 6.96 (s, 1H, NH=), 7.07–7.10 (m, 2H, Ar), 7.33 (s, 1H, NH-Ph), 7.43–7.46 (m, 1H, Ar), 7.52–7.61 (m, 4H, H-3, Ar), 7.84–7.96 (m, 4H, H-6, Ar, N*H*-NH-Ph), 8.25 (d, 1H, Ar), 9.05 (s, 1H, NHSO_2_) ppm; ^13^C NMR (50 MHz, DMSO-*d*_*6*_): δ = 19.28, 34.82, 112.92, 119.97, 124.50, 125.82, 126.24, 126.60, 128.22, 128.41, 128.80, 128.89, 129.06, 130.73, 131.71, 132.00, 132.24, 133.69, 136.19, 136.72, 140.13, 148.13, 159.19 ppm.

#### *1*-*[4*-*Chloro*-*5*-*methyl*-*2*-*(naphthalen*-*1*-*ylmethylthio)phenylsulfonyl]*-*3*-*(4*-*chlorophenylamino)guanidine* (**22**, C_25_H_22_Cl_2_N_4_O_2_S_2_)

Starting from 1.54 g **8** and 0.63 g 4-chlorophenylhydrazine hydrochloride in 10 cm^3^ dry toluene for 3 h, the title compound **22** was obtained. Yield: 1.34 g (70 %); m.p.: 148–149 °C; TLC: *R*_f_ = 0.68 (CHCl_3_:pentane:acetone = 1:1:0.5); IR (KBr): $\bar{v}$ = 3,448, 3,318 (NH), 2,923 (CH_3_, CH_2_), 1,340, 1,140 (SO_2_) cm^−1^; ^1^H NMR (200 MHz, DMSO-*d*_*6*_): δ = 2.33 (s, 3H, CH_3_), 4.80 (s, 2H, S–CH_2_), 6.60 (d, 2H, Ar), 6.98 (s, 1H, NH=), 7.07 (d, 2H, Ar), 7.35–7.64 (m, 6H, Ar, NH), 7.84–8.10 (m, 4H, Ar, NH), 8.50 (d, 1H, Ar), 9.05 (s, 1H, NHSO_2_) ppm; ^13^C NMR (50 MHz, DMSO-*d*_*6*_): δ = 19.27, 34.77, 114.35, 123.23, 124.51, 125.81, 126.25, 126.60, 128.27, 128.45, 128.78, 130.72, 131.73, 131.98, 132.22, 133.68, 136.22, 136.77, 139.97, 147.18, 159.01 ppm.

#### *1*-*[4*-*Chloro*-*5*-*methyl*-*2*-*(naphthalen*-*1*-*ylmethylthio)phenylsulfonyl]*-*3*-*(4*-*methylphenylsulfonylamino)guanidine* (**23**, C_26_H_25_ClN_4_O_4_S_3_)

Starting from 1.54 g **8**, 0.65 g *p*-toluenesulfonyl hydrazide, and 0.66 g PTSA in 70 cm^3^ dry toluene for 2.5 h, the title compound **23** was obtained. Yield: 1.61 g (78 %); m.p.: 203–206 °C; TLC: *R*_f_ = 0.32 (CHCl_3_:pentane:acetone = 1:1:0.5); IR (KBr): $\bar{v}$ = 3,475, 3,370, 3,310 (NH), 2,923 (CH_3_, CH_2_), 1633 (C=N), 1,339, 1,172, 1,146 (SO_2_) cm^−1^; ^1^H NMR (200 MHz, DMSO-*d*_*6*_): δ = 2.33 (s, 3H, CH_3_), 2.36 (s, 3H, CH_3_), 4.74 (s, 2H, S–CH_2_), 7.20 (brs, 1H, NH), 7.31 (d, 2H, Ar tosyl), 7.44 (s, 1H, NH), 7.48–7.74 (m, 6H, Ar naphth, tosyl), 7.66 (s, 1H, H-3), 7.82 (s, 1H, H-6), 7.84–8.02 (m, 2H, Ar naphth), 8.24 (d, 1H, Ar naphth), 9.20 (s, 1H, SO_2_NH), 9.86 (s, 1H, SO_2_NH) ppm; ^13^C NMR (50 MHz, DMSO-*d*_*6*_): δ = 19.28, 21.33, 34.97, 124.49, 125.79, 126.24, 126.58, 128.12, 128.22, 128.41, 128.80, 128.96, 129.86, 130.57, 131.67, 132.07, 132.46, 133.68, 134.65, 135.97, 136.79, 139.94, 144.13, 158.45 ppm.

#### *1*-*[4*-*Chloro*-*2*-*(1,2*-*dihydro*-*2*-*oxoquinolin*-*4*-*ylmethylthio)*-*5*-*methylphenylsulfonyl]*-*3*-*(phenylamino)guanidine* (**24**, C_24_H_22_ClN_5_O_3_S_2_)

Starting from 1.6 g **9** and 0.51 g phenylhydrazine hydrochloride in 15 cm^3^ dry toluene for 8 h, the title compound **24** was obtained. Yield: 1.06 g (58 %); m.p.: 171–173 °C; TLC: *R*_f_ = 0.70 (CHCl_3_:MeOH = 16:3); IR (KBr): $\bar{v}$ = 3,343 (NH), 2,922 (CH_3_, CH_2_), 1,663 (CO), 1,386, 1,143 (SO_2_) cm^−1^; ^1^H NMR (500 MHz, DMSO-*d*_*6*_): δ = 2.31 (s, 3H, CH_3_), 4.62 (s, 2H, S–CH_2_), 6.66 (d, 2H, Ar), 6.69 (s, 1H, Ar), 6.76 (t, 1H, Ar), 7.00 (s, 1H, NH =), 7.13 (t, 2H, Ar), 7.22 (t, 1H, Ar), 7.32 (d, 1H, Ar), 7.39 (s, 1H, NH-Ph), 7.50 (t, 1H, Ar), 7.52 (s, 1H, H-3), 7.88 (s, 1H, H-6), 7.90 (s, 1H, N*H*-NH-Ph), 7.94 (d, 1H, Ar), 9.14 (s, 1H, NHSO_2_), 11.78 (s, 1H, NH-quinol) ppm; ^13^C NMR (50 MHz, DMSO-*d*_*6*_): δ = 19.03, 33.16, 112.69, 115.69, 118.31, 119.75, 121.67, 121.83, 125.01, 128.46, 128.85, 130.51, 130.61, 132.64, 134.39, 136.49, 138.90, 140.48, 146.08, 147.88, 158.91, 161.55 ppm.

#### *1*-*[4*-*Chloro*-*2*-*(2,3*-*dihydrobenzo[b][1,4]dioxin*-*2*-*ylmethylthio)*-*5*-*methylphenylsulfonyl]*-*3*-*(phenylamino)guanidine* (**25**, C_23_H_23_ClN_4_O_4_S_2_)

Starting from 1.57 g **10** and 0.53 g phenylhydrazine hydrochloride in 8 cm^3^ dry toluene for 1 h, the title compound **25** was obtained. Yield: 1.12 g (62 %); m.p.: 175–177 °C; TLC: *R*_f_ = 0.70 (CHCl_3_:pentane:acetone = 1:1:0.5); IR (KBr): $\bar{v}$ = 3,442 (NH), 2,923 (CH_3_, CH_2_), 1,399, 1,145 (SO_2_) cm^−1^; ^1^H NMR (500 MHz, DMSO-*d*_*6*_): δ = 2.33 (s, 3H, CH_3_), 3.32-3.48 (m, 2H, S–CH_2_), 4.06–4.10 (m, 1H, CH–O), 4.37 (d, 2H, CH_2_–O), 6.68 (d, 2H, Ar), 6.77 (t, 1H, Ar), 6.81–6.87 (m, 4H, Ar), 7.03 (s, 1H, NH=), 7.15 (t, 2H, Ar), 7.41 (s, 1H, NH-Ph), 7.64 (s, 1H, H-3), 7.88 (s, 1H, H-6), 7.90 (s, 1H, N*H*-NH-Ph), 9.09 (s, 1H, NHSO_2_) ppm; ^13^C NMR (50 MHz, DMSO-*d*_*6*_): δ = 19.29, 33.19, 66.32, 72.02, 112.96, 117.23, 117.41, 120.03, 121.66, 121.83, 128.87, 129.11, 130.74, 132.78, 134.89, 136.86, 140.95, 142.77, 143.10, 148.19, 159.23 ppm.

### General procedure for the preparation of 4-chloro-5-methyl-2-(R^1^-methylthio)-*N*-(1-R^2^-4,5-dihydro-5-oxo-1H-1,2,4-triazol-3-yl)benzenesulfonamide derivatives** 26**–**40**

The reaction was carried out in a two-neck round-bottom flask (capacity 5 cm^3^) with drying tube protection. To the cooled (0 °C) mixture of the corresponding aminoguanidines **11**–**25** (1 mmol) in dry THF, 0.46 cm^3^ TsNCO (3 mmol) was added dropwise, and the reaction mixture was stirred at room temperature for 1 h, then at reflux for 8–36 h. After cooling (0 °C, overnight) the reaction product was isolated in precipitate state (**27**–**31**, **34**–**40**) or in oil form (**26**, **32**, and **33**) and purified by crystallization from ethanol (**26**-**31**, **33**–**39**), ethyl acetate (**32**), or acetonitrile (**40**).

#### *2*-*(Benzylthio)*-*4*-*chloro*-*N*-*(4,5*-*dihydro*-*5*-*oxo*-*1H*-*1,2,4*-*triazol*-*3*-*yl)*-*5*-*methylbenzenesulfonamide* (**26**, C_16_H_15_ClN_4_O_3_S_2_)

Starting from 0.385 g **11** (1 mmol) in 1.5 cm^3^ THF, the reaction mixture was refluxed for 8 h. After cooling to room temperature, the oily solution was treated with 30 cm^3^ diethyl ether. The ether solution was decanted from the solid, evaporated to dryness, and the residue crystallized from ethanol to obtain 0.065 g (16 %) of **26**. The deposit after decantation was treated with 20 cm^3^ diethyl ether, filtered off, and purified by crystallization from ethanol to give 0.123 g (30 %) as a second fraction of **26**. M.p.: 278–279 °C; TLC: *R*_f_ = 0.44 (benzene:EtOH = 2:1); IR (KBr): $\bar{v}$ = 3,346 (NH), 2,929 (CH_3_, CH_2_), 1,688 (CO), 1,355, 1,161 (SO_2_) cm^−1^; ^1^H NMR (500 MHz, DMSO-*d*_*6*_): δ = 2.32 (s, 3H, CH_3_), 4.36 (s, 2H, S–CH_2_), 7.26 (t, 1H, Ar), 7.32 (t, 2H, Ar), 7.43 (d, 2H, Ar), 7.54 (s, 1H, H-3), 7.86 (s, 1H, H-6), 11.19 (s, 1H, NH), 11.50 (s, 1H, NH) ppm; ^13^C NMR (50 MHz, DMSO-*d*_*6*_): δ = 19.29, 36.81, 125.89, 127.63, 128.73, 129.49, 132.02, 132.78, 136.05, 136.29, 138.29, 154.50 ppm; LC–MS (IT-TOF): *m*/*z* = 410 (M^+^), *t*_R_ = 5 min.

#### *2*-*(Benzylthio)*-*4*-*chloro*-*N*-*(4,5*-*dihydro*-*1*-*methyl*-*5*-*oxo*-*1H*-*1,2,4*-*triazol*-*3*-*yl)*-*5*-*methylbenzenesulfonamide* (**27**, C_17_H_17_ClN_4_O_3_S_2_)

Starting from 0.399 g **12** (1 mmol) in 1.5 cm^3^ THF, the reaction mixture was refluxed for 8 h. The product was purified to give 0.263 g (62 %) of **27**. M.p.: 226–228 °C; TLC: *R*_f_ = 0.22 (benzene:EtOH = 2:1); IR (KBr): $\bar{v}$ = 3,102 (NH), 2,924 (CH_3_, CH_2_), 1,764 (CO), 1,319, 1,131 (SO_2_) cm^−1^; ^1^H NMR (500 MHz, DMSO-*d*_*6*_): δ = 2.31 (s, 3H, CH_3_), 3.19 (s, 3H, CH_3_), 4.32 (s, 2H, S–CH_2_), 7.25 (t, 1H, Ar), 7.31 (t, 2H, Ar), 7.37 (d, 2H, Ar), 7.51 (s, 1H, H-3), 7.94 (s, 1H, H-6), 11.75 (brs, 1H, NH) ppm; ^13^C NMR (50 MHz, DMSO-*d*_*6*_): δ = 19.21, 32.66, 36.38, 127.51, 128.04, 128.71, 129.20, 130.93, 132.18, 135.60, 136.67, 136.84, 139.58, 147.70, 152.49 ppm; LC–MS (IT-TOF): *m*/*z* = 424 (M^+^), *t*_R_ = 6 min.

#### *2*-*(Benzylthio)*-*4*-*chloro*-*N*-*(4,5*-*dihydro*-*5*-*oxo*-*1*-*phenyl*-*1H*-*1,2,4*-*triazol*-*3*-*yl)*-*5*-*methylbenzenesulfonamide* (**28**, C_22_H_19_ClN_4_O_3_S_2_)

Starting from 0.461 g **13** (1 mmol) in 1.5 cm^3^ THF, the reaction mixture was refluxed for 9 h. The product was purified to give 0.362 g (74 %) of **28**. M.p.: 212–214.5 °C; TLC: *R*_f_ = 0.61 (benzene:EtOH = 2:1); IR (KBr): $\bar{v}$ = 3,240 (NH), 2,923 (CH_3_, CH_2_), 1,702 (CO), 1,354, 1,173 (SO_2_) cm^−1^; ^1^H NMR (200 MHz, DMSO-*d*_*6*_): δ = 2.36 (s, 3H, CH_3_), 4.34 (s, 2H, S–CH_2_), 7.12–7.24 (m, 4H, Ar), 7.30–7.44 (m, 4H, Ar), 7.58 (s, 1H, H-3), 7.66 (d, 2H, Ar), 7.98 (s, 1H, H-6), 11.98 (s, 1H, NH) ppm; ^13^C NMR (50 MHz, DMSO-*d*_*6*_): δ = 19.21, 36.70, 117.66, 124.74, 127.60, 128.49, 128.63, 129.19, 129.44, 132.80, 133.21, 135.55, 135.85, 136.71, 137.91, 138.92, 139.52, 151.52 ppm; LC–MS (IT-TOF): *m*/*z* = 486 (M^+^), *t*_R_ = 13 min.

#### *2*-*(Benzylthio)*-*4*-*chloro*-*N*-*[4,5*-*dihydro*-*1*-*(4*-*nitrophenylsulfonyl)*-*5*-*oxo*-*1H*-*1,2,4*-*triazol*-*3*-*yl]*-*5*-*methylbenzenesulfonamide* (**29**, C_22_H_18_ClN_5_O_7_S_3_)

Starting from 0.596 g **14** (1 mmol) in 2 cm^3^ THF, the reaction mixture was refluxed for 9 h. The product was purified to give 0.30 g (50 %) of **29**. M.p.: 211–214 °C; TLC: *R*_f_ = 0.59 (benzene:ethanol = 2:1); IR (KBr): $\bar{v}$ = 3,429, 3,269 (NH), 1,764 (CO), 1,536, 1,350 (NO_2_), 1,403, 1,391, 1,184, 1,167 (SO_2_) cm^−1^; ^1^H NMR (200 MHz, DMSO-*d*_*6*_): δ = 2.32 (s, 3H, CH_3_), 4.28 (s, 2H, S–CH_2_), 7.10–7.21 (m, 3H, Ar), 7.30 (d, 2H, Ar), 7.50 (s, 1H, H-3), 7.78 (d, 2H, *J* = 8.8 Hz, Ar), 7.89 (s, 1H, H-6), 8.24 (d, 2H, *J* = 8.8 Hz, Ar) ppm; ^13^C NMR (50 MHz, DMSO-*d*_*6*_): δ = 19.19, 36.41, 124.92, 127.42, 128.30, 128.40, 128.47, 128.82, 129.16, 132.48, 133.47, 135.98, 136.15, 138.37, 141.76, 144.95, 150.91, 151.71 ppm; LC–MS (IT-TOF): *m*/*z* = 596 (M^+^), *t*_R_ = 15 min.

#### *2*-*(Benzylthio)*-*4*-*chloro*-*N*-*[4,5*-*dihydro*-*1*-*(4*-*methylphenylsulfonyl)*-*5*-*oxo*-*1H*-*1,2,4*-*triazol*-*3*-*yl]*-*5*-*methylbenzenesulfonamide* (**30**, C_23_H_21_ClN_4_O_5_S_3_)

Starting from 0.539 g **15** (1 mmol) in 1.5 cm^3^ THF, the reaction mixture was refluxed for 8 h. The product was purified to give 0.405 g (72 %) of **30**. M.p.: 202–204 °C; TLC: *R*_f_ = 0.60 (benzene:EtOH = 2:1); IR (KBr): $\bar{v}$ = 3,371 (NH), 2,922 (CH_3_, CH_2_), 1,755 (CO), 1,387, 1,191, 1,176 (SO_2_) cm^−1^; ^1^H NMR (500 MHz, DMSO-*d*_*6*_): δ = 2.34 (s, 3H, CH_3_), 2.37 (s, 3H, CH_3_), 4.28 (s, 2H, S–CH_2_), 7.20 (t, 1H, Ar), 7.26 (d, 2H, Ar), 7.33 (d, 2H, Ar), 7.36 (d, 2H, Ar), 7.51 (d, 2H, Ar), 7.54 (s, 1H, H-3), 7.89 (s, 1H, H-6), 11.93 (s, 1H, NH) ppm; ^13^C NMR (50 MHz, DMSO-*d*_*6*_): δ = 19.23, 21.46, 36.67, 127.32, 127.60, 128.52, 128.66, 129.32, 130.18, 132.69, 133.33, 133.95, 135.42, 135.86, 136.42, 138.77, 143.34, 145.82, 151.67 ppm; LC–MS (IT-TOF): *m*/*z* = 564 (M^+^), *t*_R_ = 12 min.

#### *4*-*Chloro*-*N*-*[4,5*-*dihydro*-*1*-*(4*-*methylphenyl)*-*5*-*oxo*-*1H*-*1,2,4*-*triazol*-*3*-*yl]*-*2*-*(ethoxycarbonylmethylthio)*-*5*-*methylbenzenesulfonamide* (**31**, C_20_H_21_ClN_4_O_5_S_2_)

Starting from 0.471 g **16** (1 mmol) in 1.5 cm^3^ THF, the reaction mixture was refluxed for 8 h. The precipitate of by-products was filtered off. The filtrate was evaporated to dryness under reduced pressure and purified to give 0.343 g (69 %) of **31**. M.p.: 190–191 °C; TLC: *R*_f_ = 0.42 (benzene:EtOH = 2:1); IR (KBr): $\bar{v}$ = 3,255 (NH), 2,978, 2,801 (CH_3_, CH_2_), 1,726 (CO), 1,336, 1,171 (SO_2_) cm^−1^; ^1^H NMR (200 MHz, DMSO-*d*_*6*_): δ = 1.04 (t, 3H, CH_3_), 2.26 (s, 3H, CH_3_), 2.38 (s, 3H, CH_3_), 3.97-4.08 (m, 4H, S–CH_2_, CH_2_), 7.18 (d, 2H, Ar), 7.52 (d, 2H, Ar), 7.60 (s, 1H, H-3), 8.01 (s, 1H, H-6), 11.98 (s, 1H, NH) ppm; ^13^C NMR (50 MHz, DMSO-*d*_*6*_): δ = 14.10, 19.23, 20.66, 35.15, 61.43, 117.76, 129.14, 129.54, 133.20, 133.51, 133.90, 135.33, 135.51, 136.11, 138.90, 139.23, 151.40, 168.89 ppm; LC–MS (IT-TOF): *m*/*z* = 496 (M^+^), *t*_R_ = 12 min.

#### *4*-*Chloro*-*N*-*(4,5*-*dihydro*-*5*-*oxo*-*1*-*phenyl*-*1H*-*1,2,4*-*triazol*-*3*-*yl)*-*2*-*(1,3*-*dioxolan*-*2*-*ylmethylthio)*-*5*-*methylbenzenesulfonamide* (**32**, C_19_H_19_ClN_4_O_5_S_2_)

Starting from 0.458 g **17** (1 mmol) in 3 cm^3^ THF, the reaction mixture was refluxed for 5 h. After cooling to room temperature, the oily residue was treated with diethyl ether to obtain a white solid. The crude product was purified to give 0.159 g (33 %) of **32**. M.p.: 214–217 °C; TLC: *R*_f_ = 0.36 (benzene:EtOH = 2:1); IR (KBr): $\bar{v}$ = 3,414 (NH), 2,972 (CH_3_, CH_2_), 1,716 (CO), 1,382, 1,165 (SO_2_) cm^−1^; ^1^H NMR (500 MHz, DMSO-*d*_*6*_): δ = 2.37 (s, 3H, CH_3_), 3.28 (d, 2H, S–CH_2_), 3.71-3.75 (m, 2H, CH_2_–O), 3.83–3.88 (m, 2H, CH_2_–O), 5.05 (t, 1H, CH–O), 7.13 (t, 1H, Ar), 7.36 (t, 2H, Ar), 7.64 (d, 2H, Ar), 7.68 (s, 1H, H-3), 7.97 (s, 1H, H-6), 11.97 (s, 1H, NH) ppm; ^13^C NMR (50 MHz, DMSO-*d*_*6*_): δ = 19.22, 36.70, 64.96, 102.10, 117.69, 124.73, 129.18, 129.31, 129.83, 132.98, 136.13, 136.74, 137.89, 138.87, 139.64, 151.54 ppm; LC–MS (IT-TOF): *m*/*z* = 482 (M^+^), *t*_R_ = 9 min.

#### *4*-*Chloro*-*N*-*(4,5*-*dihydro*-*5*-*oxo*-*1*-*phenyl*-*1H*-*1,2,4*-*triazol*-*3*-*yl)*-*5*-*methyl*-*2*-*[3*-*(trifluoromethyl)benzylthio]benzenesulfonamide* (**33**, C_23_H_18_ClF_3_N_4_O_3_S_2_)

Starting from 0.506 g **18** (1 mmol) in 1 cm^3^ THF, the reaction mixture was refluxed for 9 h. After cooling to room temperature, the oily residue was treated with diethyl ether to obtain a white solid. The crude product was purified to give 0.210 g (38 %) of **33**. M.p.: 195–198 °C; TLC: *R*_f_ = 0.45 (benzene:EtOH = 2:1); IR (KBr): $\bar{v}$ = 3,425 (NH), 2,924 (CH_3_, CH_2_), 1,702 (CO), 1,334, 1,170 (SO_2_) cm^−1^; ^1^H NMR (200 MHz, DMSO-*d*_*6*_): δ = 2.36 (s, 3H, CH_3_), 4.48 (s, 2H, S-CH_2_), 7.14 (t, 1H, Ar), 7.27–7.58 (m, 4H, Ar), 7.58 (s, 1H, H-3), 7.62–7.76 (m, 4H, Ar), 7.98 (s, 1H, H-6), 12.00 (s, 1H, NH) ppm; ^13^C NMR (50 MHz, DMSO-*d*_*6*_): δ = 19.20, 36.05, 117.62, 124.20, 124.29, 124.71, 126.08, 126.16, 128.93, 129.14, 129.32, 129.56, 133.24, 133.33, 133.43, 135.44, 136.33, 137.85, 138.81, 139.48, 151.49 ppm; LC–MS (IT-TOF): *m*/*z* = 554 (M^+^), *t*_R_ = 17 min.

#### *4*-*Chloro*-*N*-*[4,5*-*dihydro*-*1*-*(4*-*methylphenylsulfonyl)*-*5*-*oxo*-*1H*-*1,2,4*-*triazol*-*3*-*yl]*-*5*-*methyl*-*2*-*[3*-*(trifluoromethyl)benzylthio]benzenesulfonamide* (**34**, C_24_H_20_ClF_3_N_4_O_5_S_3_)

Starting from 0.607 g **19** (1 mmol) in 2 cm^3^ THF, the reaction mixture was refluxed for 36 h. The product was purified to give 0.443 g (71 %) of **34**. M.p.: 99–100 °C; TLC: *R*_f_ = 0.62 (benzene:EtOH = 2:1); IR (KBr): $\bar{v}$ = 3,284 (NH), 2,924 (CH_3_, CH_2_), 1,716 (CO), 1,331, 1,347, 1,170, 1,194 (SO_2_) cm^−1^; ^1^H NMR (500 MHz, DMSO-*d*_*6*_): δ = 2.32 (s, 3H, CH_3_), 2.34 (s, 3H, CH_3_), 4.38 (s, 2H, S–CH_2_), 7.30 (d, 2H, Ar), 7.43–7.52 (m, 4H, Ar), 7.55–7.69 (m, 3H, Ar), 7.86 (s, 1H, H-6), 11.90 (brs, 1H, NH) ppm; ^13^C NMR (50 MHz, DMSO-*d*_*6*_): δ = 19.24, 21.36, 36.03, 127.34, 127.79, 129.03, 129.78, 129.85, 130.14, 133.12, 133.29, 133.98, 135.36, 136.18, 136.55, 137.83, 138.61, 143.63, 144.62, 145.77, 148.09, 151.75 ppm; LC–MS (IT-TOF): *m*/*z* = 632 (M^+^), *t*_R_ = 20 min.

#### *4*-*Chloro*-*N*-*(4,5*-*dihydro*-*5*-*oxo*-*1*-*phenyl*-*1H*-*1,2,4*-*triazol*-*3*-*yl)*-*5*-*methyl*-*2*-*[4*-*(trifluoromethyl)benzylthio]benzenesulfonamide* (**35**, C_23_H_18_ClF_3_N_4_O_3_S_2_)

Starting from 0.529 g **20** (1 mmol) in 1 cm^3^ THF, the reaction mixture was refluxed for 9 h. The product was purified to give 0.29 g (52 %) of **35**. M.p.: 208–210 °C; TLC: *R*_f_ = 0.40 (benzene:EtOH = 2:1); IR (KBr): $\bar{v}$ = 3,253 (NH), 2,923 (CH_3_, CH_2_), 1,701 (CO), 1,327, 1,127 (SO_2_) cm^−1^; ^1^H NMR (500 MHz, DMSO-*d*_*6*_): δ = 2.35 (s, 3H, CH_3_), 4.46 (s, 2H, S–CH_2_), 7.14 (t, 1H, Ar), 7.36 (t, 2H, Ar), 7.50 (d, 2H, Ar), 7.56 (d, 2H, Ar), 7.59 (s, 1H, H-3), 7.63 (d, 2H, Ar), 7.97 (s, 1H, H-6), 11.99 (s, 1H, NH) ppm; ^13^C NMR (50 MHz, DMSO-*d*_*6*_): δ = 19.21, 35.92, 117.62, 124.70, 125.36, 125.43, 127.76, 129.15, 129.83, 130.11, 133.17, 133.25, 135.78, 135.97, 137.88, 138.89, 139.63, 144.53, 151.51 ppm; LC–MS (IT-TOF): *m*/*z* = 554 (M^+^), *t*_R_ = 17 min.

#### *4*-*Chloro*-*N*-*(4,5*-*dihydro*-*5*-*oxo*-*1*-*phenyl*-*1H*-*1,2,4*-*triazol*-*3*-*yl)*-*5*-*methyl*-*2*-*(naphthalen*-*1*-*ylmethylthio)benzenesulfonamide* (**36**, C_26_H_21_ClN_4_O_3_S_2_)

Starting from 0.513 g **21** (1 mmol) in 1 cm^3^ THF, the reaction mixture was refluxed for 9 h. After cooling to room temperature, the reaction mixture was treated with petroleum ether to obtain a white solid. The crude product was purified to give 0.166 g (31 %) of **36**. M.p.: 214–216 °C; TLC: *R*_f_ = 0.55 (CHCl_3_:MeOH = 16:3); IR (KBr): $\bar{v}$ = 3,258 (NH), 2,922 (CH_3_, CH_2_), 1,720 (CO), 1,349, 1,168 (SO_2_) cm^−1^; ^1^H NMR (500 MHz, DMSO-*d*_*6*_): δ = 2.39 (s, 3H, CH_3_), 4.80 (s, 2H, S–CH_2_), 7.15 (t, 1H, Ar), 7.32–7.42 (m, 3H, Ar), 7.51–7.53 (m, 3H, Ar), 7.62–7.73 (m, 3H, H-3, Ar), 7.83 (d, 1H, Ar), 7.92 (d, 1H, Ar), 8.00 (s, 1H, H-6), 8.20 (d, 1H, Ar), 11.90 (s, 1H, NH) ppm; ^13^C NMR (50 MHz, DMSO-*d*_*6*_): δ = 19.26, 35.02, 117.70, 124.41, 124.78, 125.69, 126.25, 126.62, 128.44, 128.58, 128.78, 129.21, 129.56, 131.43, 131.61, 132.95, 133.11, 133.64, 135.66, 137.08, 137.91, 138.95, 139.51, 151.50 ppm; LC–MS (IT-TOF): *m*/*z* = 536 (M^+^), *t*_R_ = 18 min.

#### *4*-*Chloro*-*N*-*[1*-*(4*-*chlorophenyl)*-*4,5*-*dihydro*-*5*-*oxo*-*1H*-*1,2,4*-*triazol*-*3*-*yl]*-*5*-*methyl*-*2*-*(naphthalen*-*1*-*ylmethylthio)benzenesulfonamide* (**37**, C_26_H_20_Cl_2_N_4_O_3_S_2_)

Starting from 0.545 g **22** (1 mmol) in 2 cm^3^ THF, the reaction mixture was refluxed for 9 h. The product was purified to give 0.224 g (39 %) of **37**. M.p.: 205–206 °C; TLC: *R*_f_ = 0.59 (benzene:EtOH = 2:1); IR (KBr): $\bar{v}$ = 3,251 (NH), 2,924 (CH_3_, CH_2_), 1,722 (C=O), 1,352, 1,166 (SO_2_) cm^−1^; ^1^H NMR (200 MHz, DMSO-*d*_*6*_): δ = 2.39 (s, 3H, CH_3_), 4.81 (s, 2H, S-CH_2_), 7.31–7.58 (m, 6H, Ar), 7.62–7.72 (m, 3H, Ar), 7.80–7.94 (m, 2H, Ar), 8.01 (s, 1H, H-6), 8.20 (d, 1H, Ar), 11.99 (s, 1H, NH) ppm; ^13^C NMR (50 MHz, DMSO-*d*_*6*_): δ = 19.27, 34.99, 119.09, 124.41, 125.89, 126.24, 126.61, 128.41, 128.56, 128.77, 129.17, 129.56, 131.43, 131.60, 132.96, 133.09, 133.64, 135.59, 136.76, 137.08, 138.98, 139.92, 142.10, 151.42 ppm; LC–MS (IT-TOF): *m*/*z* = 570 (M^+^), *t*_R_ = 22 min.

#### *4*-*Chloro*-*N*-*[4,5*-*dihydro*-*1*-*(4*-*methylphenylsulfonyl)*-*5*-*oxo*-*1H*-*1,2,4*-*triazol*-*3*-*yl]*-*5*-*methyl*-*2*-*(naphthalen*-*1*-*ylmethylthio)benzenesulfonamide* (**38**, C_27_H_23_ClN_4_O_5_S_3_)

Starting from 0.589 g **23** (1 mmol) in 2 cm^3^ THF, the reaction mixture was refluxed for 11 h. The product was purified to give 0.438 g (70 %) of **38**. M.p.: 118–120 °C; TLC: *R*_f_ = 0.60 (benzene:EtOH = 2:1); IR (KBr): $\bar{v}$ = 3,530 (NH), 2,973 (CH_3_, CH_2_), 1,726 (CO), 1,388, 1,370, 1,193, 1,177 (SO_2_) cm^−1^; ^1^H NMR (500 MHz, DMSO-*d*_*6*_): δ = 2.31 (s, 3H, CH_3_), 2.37 (s, 3H, CH_3_), 4.70 (s, 2H, S–CH_2_), 7.32 (d, 2H, Ar), 7.38 (t, 1H, Ar), 7.48–7.53 (m, 5H, Ar), 7.63 (s, 1H, H-3), 7.84 (d, 1H, Ar), 7.90 (s, 1H, H-6), 7.94 (d, 1H, Ar), 8.08 (d, 1H, Ar), 11.90 (brs, 1H, NH) ppm; ^13^C NMR (50 MHz, DMSO-*d*_*6*_): δ = 19.29, 21.42, 35.06, 124.28, 125.76, 126.27, 126.64, 127.37, 128.27, 128.56, 128.79, 128.96, 130.19, 131.38, 131.59, 132.90, 133.27, 133.64, 133.91, 135.68, 136.75, 138.77, 143.56, 145.85, 151.78 ppm; LC–MS (IT-TOF): *m*/*z* = 614 (M^+^), *t*_R_ = 23 min.

#### *4*-*Chloro*-*N*-*(4,5*-*dihydro*-*5*-*oxo*-*1*-*phenyl*-*1H*-*1,2,4*-*triazol*-*3*-*yl)*-*2*-*(1,2*-*dihydro*-*2*-*oxoquinolin*-*4*-*ylmethylthio)*-*5*-*methylbenzenesulfonamide* (**39**, C_25_H_20_ClN_5_O_4_S_2_)

Starting from 0.528 g **24** (1 mmol) in 3 cm^3^ THF, the reaction mixture was refluxed for 9 h. The product was purified to give 0.161 g (29 %) of **39**. M.p.: 185–188 °C; TLC: *R*_f_ = 0.12 (CHCl_3_:MeOH = 16:3), *R*_f_ = 0.19 (CHCl_3_:MeCN:AcOH = 2:1:0.05); IR (KBr): $\bar{v}$ = 3,467 (NH), 2,923 (CH_3_, CH_2_), 1,692, 1,655 (CO), 1,383, 1,127 (SO_2_) cm^−1^; ^1^H NMR (500 MHz, DMSO-*d*_*6*_): δ = 2.37 (s, 3H, CH_3_), 4.59 (s, 2H, S–CH_2_), 6.58 (s, 1H, Ar), 7.10 (t, 1H, Ar), 7.16 (t, 1H, Ar), 7.30 (d, 1H, Ar), 7.34 (t, 2H, Ar), 7.49 (t, 1H, Ar), 7.55 (s, 1H, H-3), 7.65 (d, 2H, Ar), 7.88 (d, 1H, Ar), 8.01 (s, 1H, H-6), 11.73 (s, 2H, NH-quinolin, NH-triazolone) ppm; ^13^C NMR (50 MHz, DMSO-*d*_*6*_): δ = 19.27, 33.74, 115.88, 117.75, 118.29, 121.90, 122.48, 124.75, 125.28, 129.17, 129.46, 130.83, 133.17, 133.53, 135.51, 136.33, 137.84, 138.90, 139.23, 139.47, 145.59, 151.51, 161.49 ppm; LC–MS (IT-TOF): *m*/*z* = 554 (M^+^), *t*_R_ = 17 min.

#### *4*-*Chloro*-*2*-*(2,3*-*dihydrobenzo[b][1,4]dioxin*-*2*-*ylmethylthio)*-*N*-*(4,5*-*dihydro*-*5*-*oxo*-*1*-*phenyl*-*1H*-*1,2,4*-*triazol*-*3*-*yl)*-*5*-*methylbenzenesulfonamide* (**40**, C_24_H_21_ClN_4_O_5_S_2_)

Starting from 0.519 g **25** (1 mmol) in 3 cm^3^ THF, the reaction mixture was refluxed for 9 h. The product was purified to give 0.262 g (48 %) of **40**. M.p.: 185–188 °C; TLC: *R*_f_ = 0.53 (benzene:ethanol = 2:1); IR (KBr): $\bar{v}$ = 3,311 (NH), 2,922 (CH_3_, CH_2_), 1,697 (CO), 1,334, 1,165 (SO_2_) cm^−1^; ^1^H NMR (500 MHz, DMSO-*d*_*6*_): δ = 2.38 (s, 3H, CH_3_), 3.36 (dd, 1H, S–CH_2_), 3.46 (dd, 1H, S–CH_2_), 4.00 (dd, 1H, O–CH_2_), 4.26 (dd, 1H, O–CH_2_), 4.31–4.35 (m, 1H, O–CH), 6.70–6.81 (m, 4H, Ar), 7.12 (t, 1H, Ar), 7.34 (t, 2H, Ar), 7.64 (d, 2H, Ar), 7.76 (s, 1H, H-3), 8.01 (s, 1H, H-6), 12.01 (s, 1H, NH) ppm; ^13^C NMR (50 MHz, DMSO-*d*_*6*_): δ = 19.29, 33.64, 66.26, 72.02, 117.15, 117.29, 117.69, 121.61, 121.70, 124.74, 129.14, 129.83, 133.09, 133.61, 135.83, 136.62, 137.83, 139.12, 139.50, 142.61, 142.99, 151.54 ppm; LC–MS (IT-TOF): *m*/*z* = 545 (M^+^), *t*_R_ = 21 min.

### X-ray structure determination

Experimental diffraction data were collected on a KM4 CCD kappa-geometry diffractometer (Oxford diffraction), equipped with a Sapphire2 CCD detector. An enhanced X-ray Mo Kα radiation source with a graphite monochromator was used. Determination of the unit cell and diffraction data collection were carried out at 120 K in a stream of dry nitrogen (Oxford CryoSystems). All calculations (data reduction, structure solution, and refinement) were carried out using CrysAlisPro [49] package. The structure was solved by direct methods, and all non-hydrogen atoms were refined with anisotropic thermal parameters by full-matrix least squares procedure based on *F* ^*2*^. Final refinements were carried out using the SHELX-97 package [50], run under control of WinGX program [51].

All hydrogen atoms were refined using isotropic model with *U*_iso_ *(H)* values fixed to be 1.2 times *U*_eq_ of C atoms for CH and CH_2_ and 1.5 times *U*_eq_ for CH_3_. Bond lengths C–H were fixed at 0.98 Å for methyl groups, and 0.95 Å for methylene and methine groups; distances N–H were set to 0.88 Å. Solvating water molecules generated an electron density peak of ca. 1.7 e Å^−3^. Because the electron density maximum is placed at a special position (½, *y*, ¼) localization of hydrogen atoms is additionally uncertain so we did not attempt to find H atoms. The occupation factor of oxygen atom O10 was refined freely to obtain 0.079. One incorrect reflection (−1 1 17) was omitted.

Crystallographic data for the structure of **31Pyr** reported in this article have been deposited with the Cambridge Crystallographic Data Center as supplementary publication no. CCDC868805. Copies of the data can be obtained free of charge on application to CCDC, 12 Union Road, Cambridge CB2 1EZ, UK [Fax: (+44) 1223-336-033; email: deposit@ccdc.cam.ac.uk].

## Electronic supplementary material

Below is the link to the electronic supplementary material.

Supplementary material 1 (PDF 157 kb)
