# Supplementary material for: Synthesis and molecular structure of novel 2-(alkylthio)-4-chloro-N-(4,5-dihydro-5-oxo-1H-1,2,4-triazol-3-yl)-5-methylbenzenesulfonamides with potential anticancer activity
Source: Monatsh Chem. 2012 Sep 25;143(12):1705–18. doi: 10.1007/s00706-012-0849-7 (PMC4495025; doi:10.1007/s00706-012-0849-7)

National Cancer Institute Developmental Therapeutics Program Dose Response Curves	NSC : D - 760560/1	SSPL:0X8U	EXP. ID:1108NS03
	Report Date:October 14, 2011	Test Date:August 15, 2011	

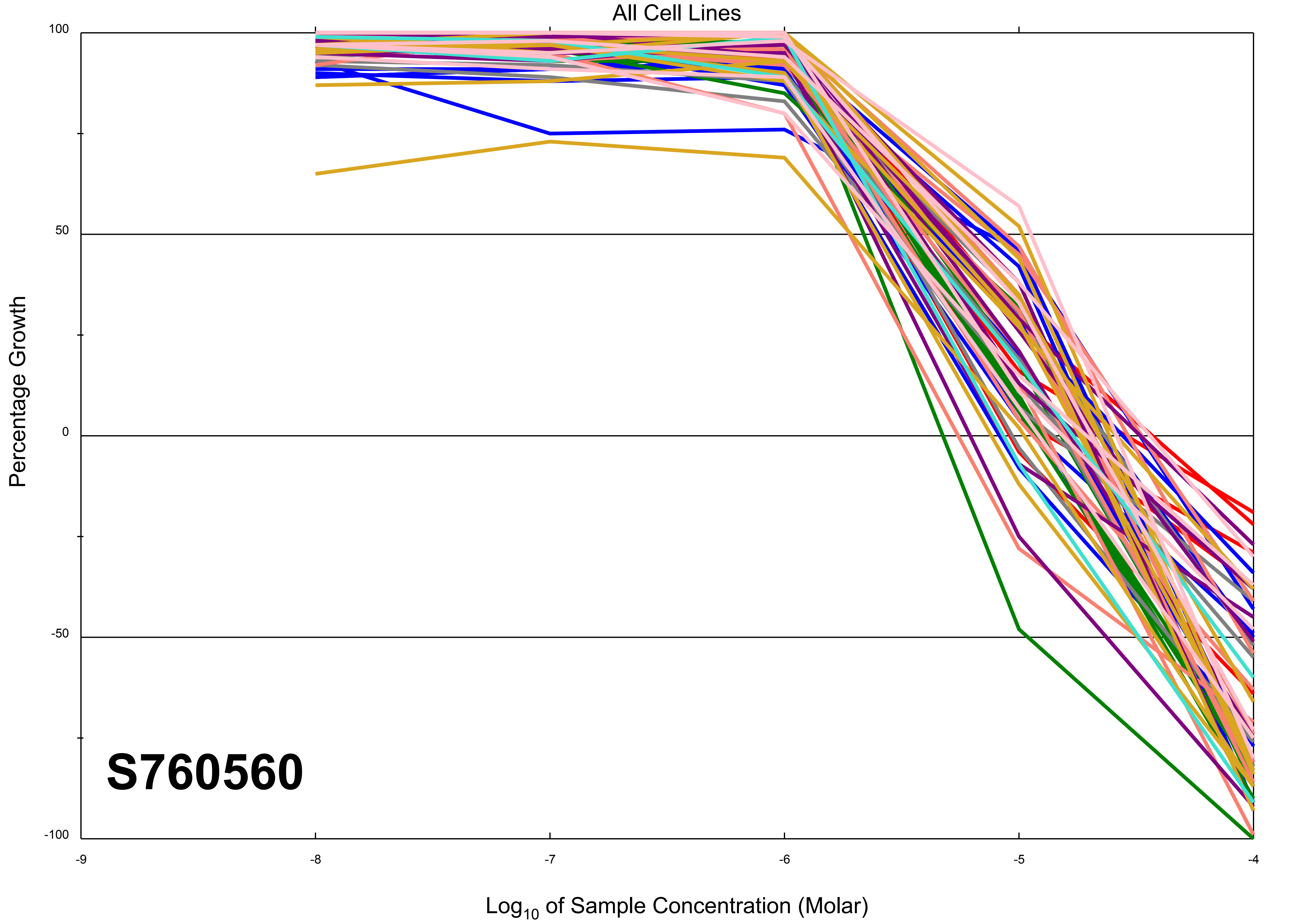

National Cancer Institute Developmental Therapeutics Program Dose Response Curves	NSC: D - 760560 / 1	SSPL: 0X8U	EXP. ID: 1108NS03
	Report Date: October 14, 2011	Test Date: August 15, 2011	

Leukemia

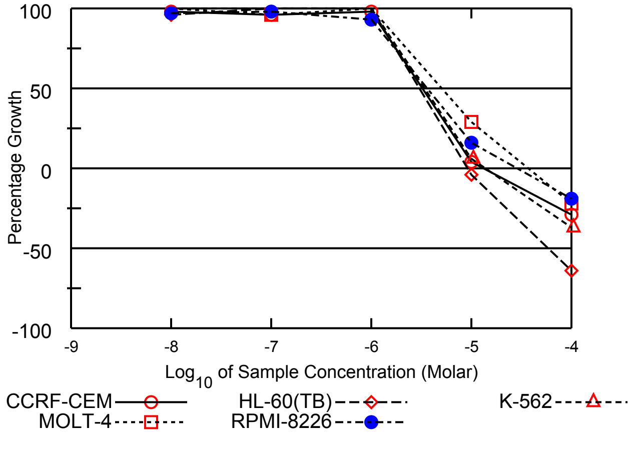

Non-Small Cell Lung Cancer

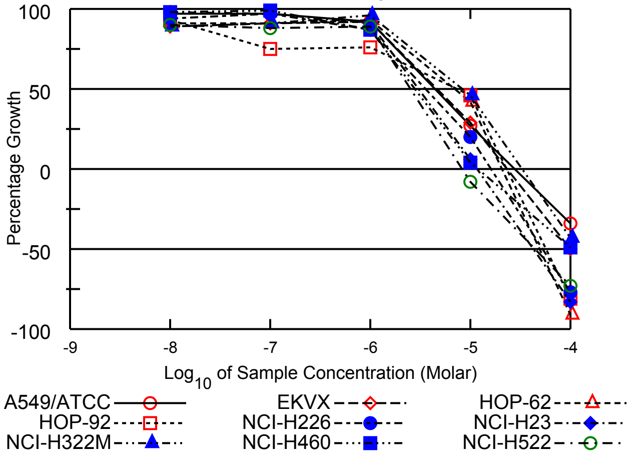

Colon Cancer

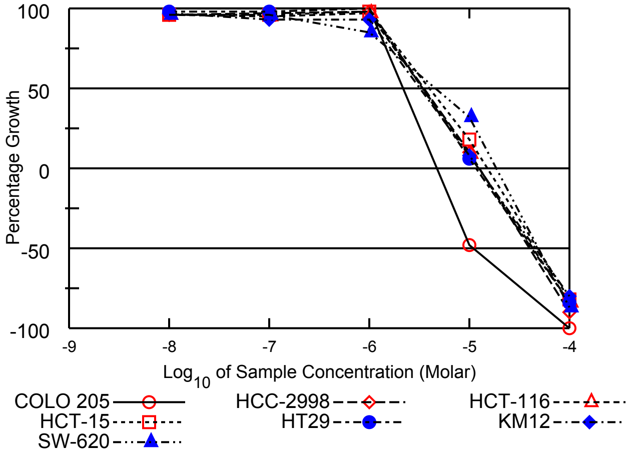

CNS Cancer

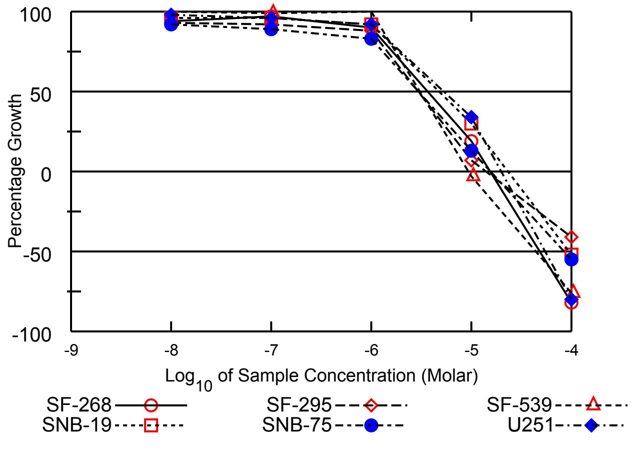

Melanoma

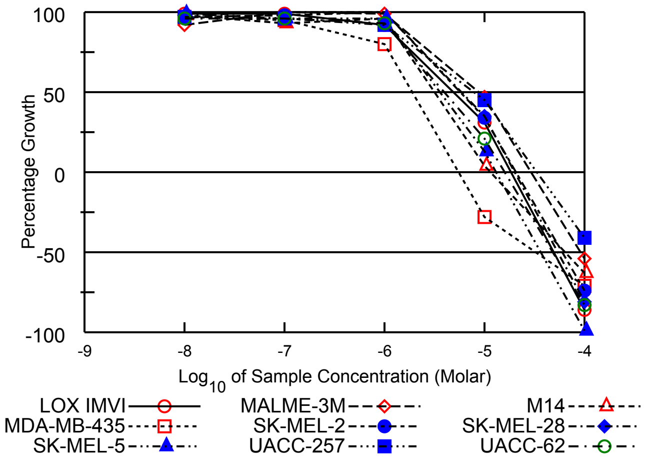

Ovarian Cancer

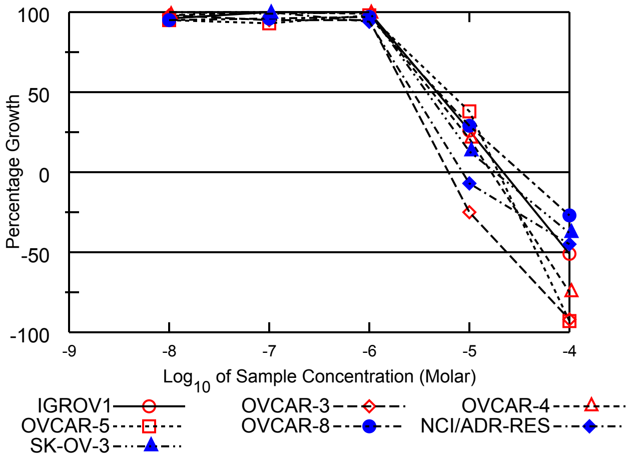

Renal Cancer

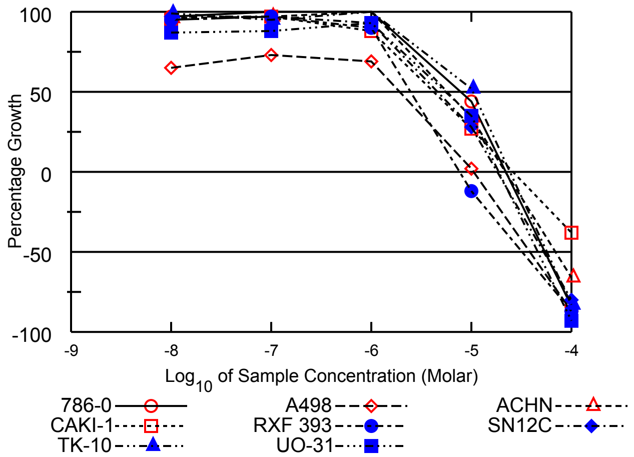

Prostate Cancer

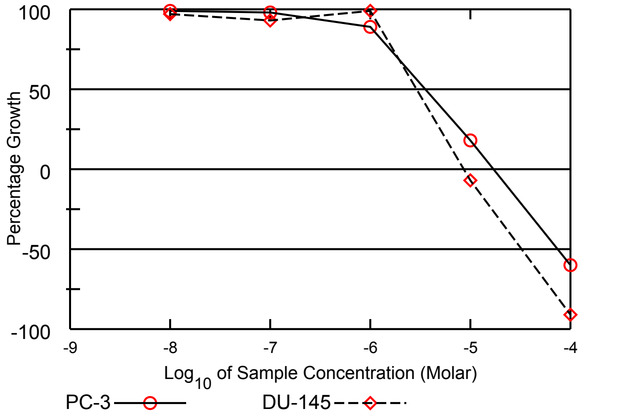

Breast Cancer

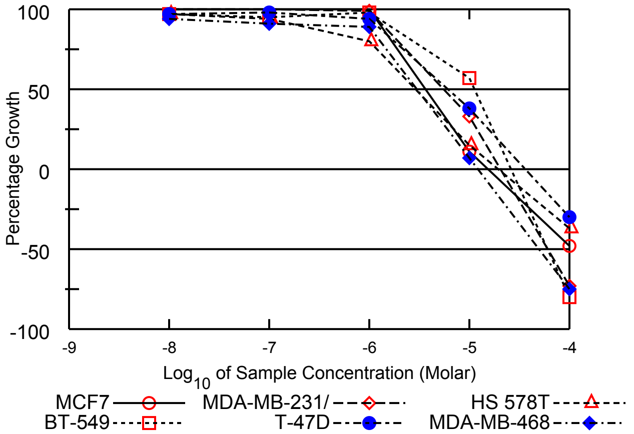

National Cancer Institute Developmental Therapeutics Program
In-Vitro Testing Results

NSC : D - 760560 / 1	Experiment ID : 1108NS03	Test Type : 08	Units : Molar
Report Date : October 14, 2011	Test Date : August 15, 2011	QNS :	MC :
COMI : JJS-296 (108488)	Stain Reagent : SRB Dual-Pass Related	SSPL : 0X8U	

			Log10 Concentration												
	Time		Mean Optical Densities					Percent Growth							
Panel/Cell Line	Zero	Ctrl	-8.0	-7.0	-6.0	-5.0	-4.0	-8.0	-7.0	-6.0	-5.0	-4.0	GI50	TGI	LC50
Leukemia															
CCRF-CEM	0.539	1.740	1.716	1.697	1.713	0.588	0.382	98	96	98	4	-29	3.23E-6	1.32E-5	> 1.00E-4
HL-60(TB)	1.054	2.762	2.692	2.783	2.804	1.015	0.380	96	101	102	-4	-64	3.12E-6	9.22E-6	5.87E-5
K-562	0.310	1.481	1.539	1.569	1.528	0.384	0.194	105	107	104	6	-37	3.57E-6	1.39E-5	> 1.00E-4
MOLT-4	1.005	2.597	2.659	2.537	2.816	1.474	0.782	104	96	114	29	-22	5.71E-6	3.72E-5	> 1.00E-4
RPMI-8226	0.840	2.154	2.109	2.134	2.065	1.045	0.680	97	98	93	16	-19	3.60E-6	2.81E-5	> 1.00E-4
Non-Small Cell Lung Cancer															
A549/ATCC	0.387	1.953	1.901	1.913	1.831	0.810	0.256	97	97	92	27	-34	4.44E-6	2.78E-5	> 1.00E-4
EKVX	0.667	1.473	1.386	1.403	1.408	0.900	0.336	89	91	92	29	-50	4.62E-6	2.33E-5	> 1.00E-4
HOP-62	0.426	1.024	0.971	0.973	0.981	0.680	0.038	91	91	93	42	-91	7.08E-6	2.08E-5	4.93E-5
HOP-92	1.022	1.431	1.400	1.329	1.335	1.211	0.193	93	75	76	46	-81	7.46E-6	2.30E-5	5.70E-5
NCI-H226	0.845	1.980	1.912	1.943	1.836	1.071	0.198	94	97	87	20	-77	3.58E-6	1.61E-5	5.30E-5
NCI-H23	0.523	1.791	1.761	1.749	1.679	0.601	0.090	98	97	91	6	-83	3.05E-6	1.17E-5	4.28E-5
NCI-H322M	0.790	1.811	1.699	1.724	1.773	1.258	0.453	89	91	96	46	-43	8.25E-6	3.29E-5	> 1.00E-4
NCI-H460	0.292	2.696	2.653	2.660	2.393	0.380	0.148	98	99	87	4	-49	2.80E-6	1.17E-5	> 1.00E-4
NCI-H522	0.846	1.913	1.811	1.786	1.792	0.781	0.226	90	88	89	-8	-73	2.52E-6	8.31E-6	4.41E-5
Colon Cancer															
COLO 205	0.324	1.222	1.257	1.246	1.261	0.170	0.001	104	103	104	-48	-100	2.28E-6	4.86E-6	1.11E-5
HCC-2998	0.635	1.898	1.849	1.864	1.874	0.763	0.066	96	97	98	10	-90	3.52E-6	1.26E-5	4.00E-5
HCT-116	0.228	1.733	1.667	1.663	1.691	0.371	0.036	96	95	97	9	-84	3.45E-6	1.26E-5	4.30E-5
HCT-15	0.400	2.485	2.403	2.400	2.442	0.786	0.071	96	96	98	18	-82	4.01E-6	1.53E-5	4.78E-5
HT29	0.305	1.613	1.590	1.584	1.644	0.384	0.049	98	98	102	6	-84	3.49E-6	1.17E-5	4.19E-5
KM12	0.285	1.511	1.471	1.421	1.425	0.389	0.058	97	93	93	8	-80	3.22E-6	1.25E-5	4.61E-5
SW-620	0.213	1.518	1.469	1.462	1.321	0.630	0.028	96	96	85	32	-87	4.56E-6	1.86E-5	4.89E-5
CNS Cancer															
SF-268	0.346	1.234	1.185	1.204	1.149	0.518	0.062	94	97	90	19	-82	3.71E-6	1.55E-5	4.82E-5
SF-295	0.627	2.261	2.151	2.123	2.072	0.738	0.373	93	92	88	7	-41	2.95E-6	1.39E-5	> 1.00E-4
SF-539	0.819	2.371	2.390	2.363	2.436	0.798	0.196	101	99	104	-3	-76	3.21E-6	9.45E-6	4.41E-5
SNB-19	0.435	1.503	1.455	1.460	1.420	0.760	0.210	96	96	92	30	-52	4.83E-6	2.35E-5	9.53E-5
SNB-75	0.432	0.921	0.883	0.865	0.836	0.495	0.196	92	89	83	13	-55	2.93E-6	1.55E-5	8.51E-5
U251	0.481	2.078	2.048	2.008	1.957	1.020	0.099	98	96	92	34	-80	5.28E-6	1.99E-5	5.49E-5
Melanoma															
LOX IMVI	0.325	1.931	1.912	1.909	1.804	0.819	0.044	99	99	92	31	-86	4.85E-6	1.83E-5	4.88E-5
MALME-3M	0.552	1.142	1.095	1.151	1.135	0.831	0.257	92	102	99	47	-54	8.86E-6	2.94E-5	9.22E-5
M14	0.356	1.272	1.252	1.205	1.237	0.394	0.134	98	93	96	4	-63	3.18E-6	1.15E-5	6.49E-5
MDA-MB-435	0.420	1.864	1.815	1.793	1.572	0.301	0.121	97	95	80	-28	-71	1.89E-6	5.47E-6	3.20E-5
SK-MEL-2	0.936	1.673	1.651	1.660	1.676	1.188	0.245	97	98	100	34	-74	5.76E-6	2.07E-5	6.02E-5
SK-MEL-28	0.403	1.050	1.040	1.065	1.065	0.630	0.078	98	102	102	35	-81	5.99E-6	2.01E-5	5.42E-5
SK-MEL-5	0.625	2.586	2.567	2.516	2.509	0.886	0.007	99	96	96	13	-99	3.60E-6	1.31E-5	3.66E-5
UACC-257	0.781	1.756	1.730	1.718	1.678	1.221	0.463	97	96	92	45	-41	7.88E-6	3.36E-5	> 1.00E-4
UACC-62	0.632	2.264	2.197	2.194	2.155	0.982	0.109	96	96	93	21	-83	4.01E-6	1.61E-5	4.85E-5
Ovarian Cancer															
IGROV1	0.439	1.585	1.538	1.610	1.617	0.740	0.215	96	102	103	26	-51	4.89E-6	2.19E-5	9.70E-5
OVCAR-3	0.540	1.536	1.517	1.491	1.486	0.405	0.043	98	95	95	-25	-92	2.37E-6	6.18E-6	2.36E-5
OVCAR-4	0.439	1.025	1.012	1.030	1.020	0.562	0.111	98	101	99	21	-75	4.25E-6	1.65E-5	5.51E-5
OVCAR-5	0.514	1.357	1.314	1.302	1.340	0.833	0.035	95	93	98	38	-93	6.28E-6	1.94E-5	4.68E-5
OVCAR-8	0.417	1.782	1.715	1.730	1.745	0.820	0.303	95	96	97	29	-27	4.98E-6	3.30E-5	> 1.00E-4
NCI/ADR-RES	0.475	1.611	1.654	1.615	1.544	0.443	0.262	104	100	94	-7	-45	2.74E-6	8.57E-6	> 1.00E-4
SK-OV-3	0.491	1.275	1.277	1.269	1.293	0.595	0.304	100	99	102	13	-38	3.87E-6	1.81E-5	> 1.00E-4
Renal Cancer															
786-0	0.790	2.238	2.190	2.248	2.301	1.423	0.128	97	101	104	44	-84	7.87E-6	2.20E-5	5.43E-5
A498	1.407	2.168	1.904	1.961	1.934	1.420	0.176	65	73	69	2	-87	1.93E-6	1.05E-5	3.80E-5
ACHN	0.341	1.480	1.439	1.441	1.486	0.731	0.115	96	97	101	34	-66	5.79E-6	2.19E-5	6.87E-5
CAKI-1	0.730	2.153	2.077	2.106	1.988	1.114	0.450	95	97	88	27	-38	4.21E-6	2.59E-5	> 1.00E-4
RXF 393	0.739	1.370	1.339	1.354	1.304	0.652	0.096	95	97	90	-12	-87	2.46E-6	7.65E-6	3.22E-5
SN12C	0.459	1.780	1.737	1.740	1.692	0.831	0.092	97	97	93	28	-80	4.62E-6	1.82E-5	5.27E-5
TK-10	0.776	1.598	1.587	1.555	1.620	1.200	0.132	99	95	103	52	-83	1.03E-5	2.42E-5	5.68E-5
UO-31	0.476	1.401	1.284	1.288	1.338	0.799	0.032	87	88	93	35	-93	5.52E-6	1.87E-5	4.59E-5
Prostate Cancer															
PC-3	0.417	1.512	1.500	1.490	1.392	0.619	0.165	99	98	89	18	-60	3.57E-6	1.71E-5	7.37E-5
DU-145	0.263	1.019	0.995	0.966	1.010	0.245	0.024	97	93	99	-7	-91	2.90E-6	8.62E-6	3.26E-5
Breast Cancer															
MCF7	0.228	1.206	1.268	1.248	1.241	0.333	0.120	106	104	104	11	-48	3.77E-6	1.53E-5	> 1.00E-4
MDA-MB-231/ATCC	0.586	1.608	1.665	1.649	1.600	0.925	0.157	106	104	99	33	-73	5.56E-6	2.05E-5	6.04E-5
HS 578T	0.895	1.865	1.839	1.810	1.668	1.045	0.560	97	94	80	15	-37	2.90E-6	1.96E-5	> 1.00E-4
BT-549	0.940	1.998	1.962	1.945	1.972	1.542	0.189	97	95	98	57	-80	1.12E-5	2.60E-5	6.04E-5
T-47D	0.550	1.504	1.479	1.486	1.449	0.913	0.383	97	98	94	38	-30	6.13E-6	3.59E-5	> 1.00E-4
MDA-MB-468	0.550	0.965	0.940	0.928	0.919	0.581	0.139	94	91	89	7	-75	3.00E-6	1.23E-5	4.99E-5

National Cancer Institute Developmental Therapeutics Program Mean Graphs	NSC : D - 760560/1	Units :Molar	SSPL :0X8U	EXP. ID :1108NS03
	Report Date :October 14, 2011		Test Date :August 15, 2011	

Panel/Cell Line	Log_{10}GI50	Log_{10}TGI	Log_{10}LC50
**Leukemia**			
CCRF-CEM	-5.49	-4.88	> -4.00
HL-60(TB)	-5.51	-5.04	-4.23
K-562	-5.45	-4.86	> -4.00
MOLT-4	-5.24	-4.43	> -4.00
RPMI-8226	-5.44	-4.55	> -4.00
**Non-Small Cell Lung Cancer**			
A549/ATCC	-5.35	-4.56	> -4.00
EKVX	-5.33	-4.63	> -4.00
HOP-62	-5.15	-4.68	-4.31
HOP-92	-5.13	-4.64	-4.24
NCI-H226	-5.45	-4.79	-4.28
NCI-H23	-5.52	-4.93	-4.37
NCI-H322M	-5.08	-4.48	> -4.00
NCI-H460	-5.55	-4.93	> -4.00
NCI-H522	-5.60	-5.08	-4.36
**Colon Cancer**			
COLO 205	-5.64	-5.31	-4.96
HCC-2998	-5.45	-4.90	-4.40
HCT-116	-5.46	-4.90	-4.37
HCT-15	-5.40	-4.82	-4.32
HT29	-5.46	-4.93	-4.38
KM12	-5.49	-4.90	-4.34
SW-620	-5.34	-4.73	-4.31
**CNS Cancer**			
SF-268	-5.43	-4.81	-4.32
SF-295	-5.53	-4.86	> -4.00
SF-539	-5.49	-5.02	-4.36
SNB-19	-5.32	-4.63	-4.02
SNB-75	-5.53	-4.81	-4.07
U251	-5.28	-4.70	-4.26
**Melanoma**			
LOX IMVI	-5.31	-4.74	-4.31
MALME-3M	-5.05	-4.53	-4.04
M14	-5.50	-4.94	-4.19
MDA-MB-435	-5.72	-5.26	-4.49
SK-MEL-2	-5.24	-4.68	-4.22
SK-MEL-28	-5.22	-4.70	-4.27
SK-MEL-5	-5.44	-4.88	-4.44
UACC-257	-5.10	-4.47	> -4.00
UACC-62	-5.40	-4.79	-4.31
**Ovarian Cancer**			
IGROV1	-5.31	-4.66	-4.01
OVCAR-3	-5.63	-5.21	-4.63
OVCAR-4	-5.37	-4.78	-4.26
OVCAR-5	-5.20	-4.71	-4.33
OVCAR-8	-5.30	-4.48	> -4.00
NCI/ADR-RES	-5.56	-5.07	> -4.00
SK-OV-3	-5.41	-4.74	> -4.00
**Renal Cancer**			
786-0	-5.10	-4.66	-4.27
A498	-5.71	-4.98	-4.42
ACHN	-5.24	-4.66	-4.16
CAKI-1	-5.38	-4.59	> -4.00
RXF 393	-5.61	-5.12	-4.49
SN12C	-5.34	-4.74	-4.28
TK-10	-4.99	-4.62	-4.25
UO-31	-5.26	-4.73	-4.34
**Prostate Cancer**			
PC-3	-5.45	-4.77	-4.13
DU-145	-5.54	-5.06	-4.49
**Breast Cancer**			
MCF7	-5.42	-4.82	> -4.00
MDA-MB-231/ATCC	-5.25	-4.69	-4.22
HS 578T	-5.54	-4.71	> -4.00
BT-549	-4.95	-4.58	-4.22
T-47D	-5.21	-4.44	> -4.00
MDA-MB-468	-5.52	-4.91	-4.30
_MID	-5.38	-4.79	-4.22
Delta	0.34	0.52	0.74
Range	0.77	0.88	0.96

GI50

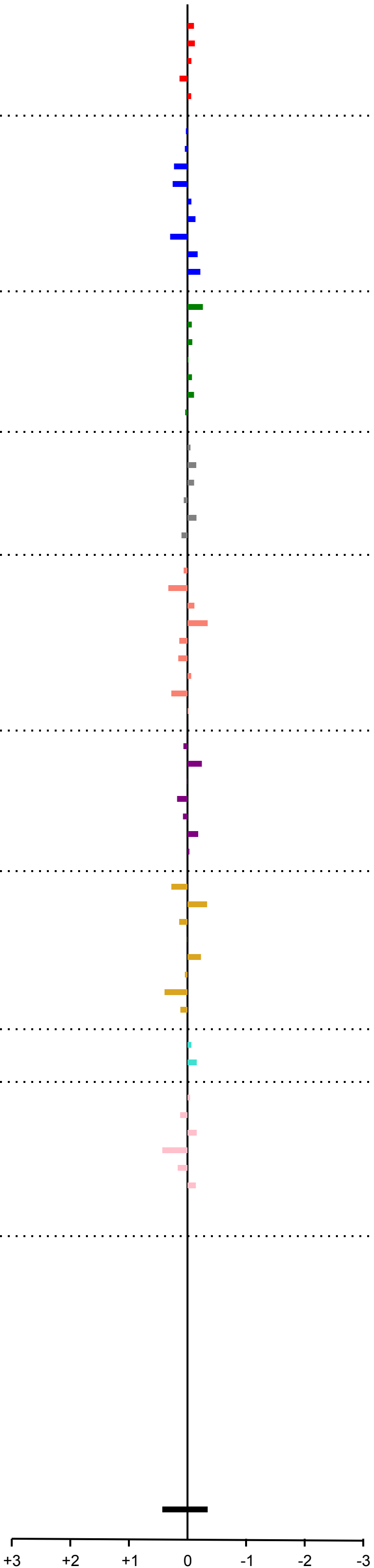

TGI

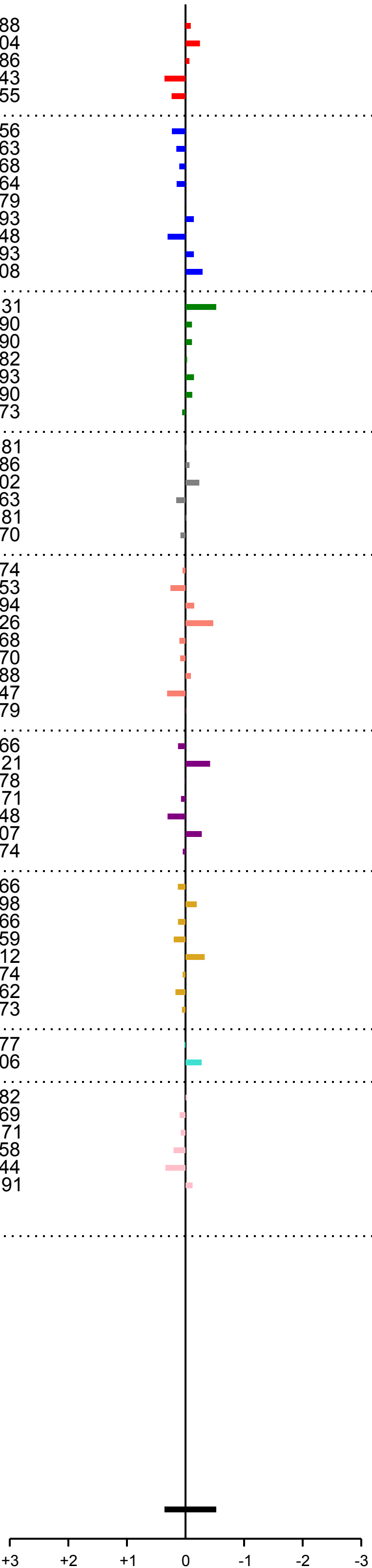

LC50

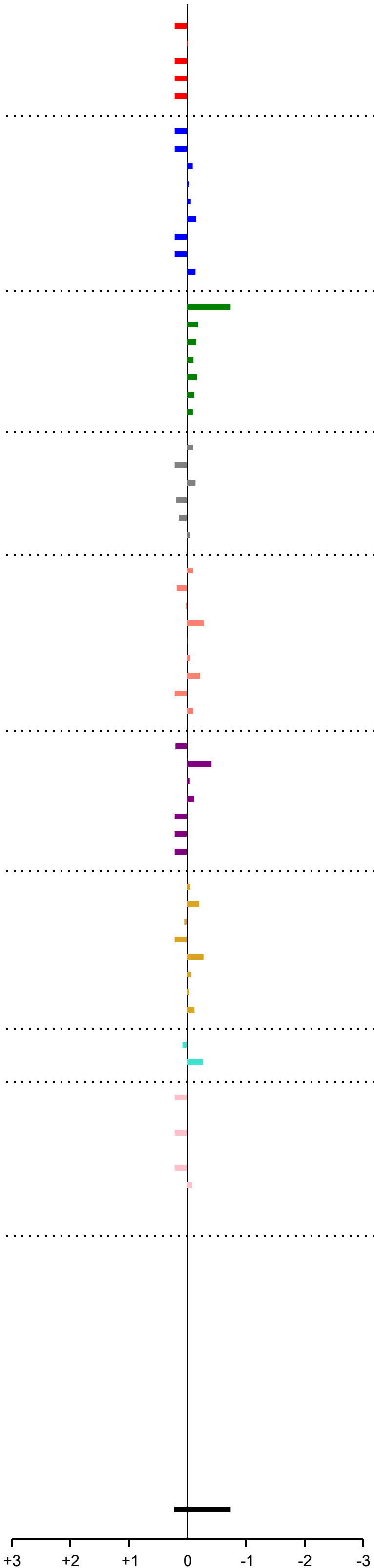

Supplement: Supplementary file 1 — Supplementary material 1 (PDF 157 kb) [file 706_2012_849_MOESM1_ESM.pdf]
